# A genomic epidemiological study shows that prevalence of antimicrobial resistance in *Enterobacterales* is associated with the livestock host, as well as antimicrobial usage

**DOI:** 10.1099/mgen.0.000630

**Published:** 2021-10-05

**Authors:** Manal AbuOun, Hannah Jones, Emma Stubberfield, Daniel Gilson, Liam P. Shaw, Alasdair T. M. Hubbard, Kevin K. Chau, Robert Sebra, Tim E. A. Peto, Derrick W. Crook, Daniel S. Read, H. Soon Gweon, A. Sarah Walker, Nicole Stoesser, Richard P. Smith, Muna F. Anjum, on behalf of the REHAB consortium

**Affiliations:** ^1^​ Department of Bacteriology, Animal and Plant Health Agency, Weybridge, Surrey, UK; ^2^​ Department of Epidemiological Sciences, Animal and Plant Health Agency, Weybridge, Surrey, UK; ^3^​ Modernising Medical Microbiology Consortium, Nuffield Department of Medicine, University of Oxford, Oxford, UK; ^4^​ Department of Tropical Disease Biology, Liverpool School of Tropical Medicine, Liverpool, UK; ^5^​ Department of Genetic and Genomic Sciences, Icahn School of Medicine at Mt Sinai, Mt Sinai, New York, USA; ^6^​ National Institute for Health Research, Health Protection Research Unit, University of Oxford in partnership with Public Health England (PHE), Oxford, UK; ^7^​ UK Centre for Ecology & Hydrology (UKCEH), Benson Lane, Crowmarsh Gifford, Wallingford, UK; ^8^​ School of Biological Sciences, University of Reading, UK

**Keywords:** antimicrobial resistance, plasmid, antimicrobial usage, livestock

## Abstract

Enterobacterales from livestock are potentially important reservoirs for antimicrobial resistance (AMR) to pass through the food chain to humans, thereby increasing the AMR burden and affecting our ability to tackle infections. In this study 168 isolates from four genera of the order *Enterobacterales *, primarily *Escherichia coli *, were purified from livestock (cattle, pigs and sheep) faeces from 14 farms in the United Kingdom. Their genomes were resolved using long- and short-read sequencing to analyse AMR genes and their genetic context, as well as to explore the relationship between AMR burden and on-farm antimicrobial usage (AMU), in the three months prior to sampling. Although *E. coli* isolates were genomically diverse, phylogenetic analysis using a core-genome SNP tree indicated pig isolates to generally be distinct from sheep isolates, with cattle isolates being intermediates. Approximately 28 % of isolates harboured AMR genes, with the greatest proportion detected in pigs, followed by cattle then sheep; pig isolates also harboured the highest number of AMR genes per isolate. Although 90 % of sequenced isolates harboured diverse plasmids, only 11 % of plasmids (*n*=58 out of 522) identified contained AMR genes, with 91 % of AMR plasmids being from pig, 9 % from cattle and none from sheep isolates; these results indicated that pigs were a principle reservoir of AMR genes harboured by plasmids and likely to be involved in their horizontal transfer. Significant associations were observed between AMU (mg kg^−1^) and AMR. As both the total and the numbers of different antimicrobial classes used on-farm increased, the risk of multi-drug resistance (MDR) in isolates rose. However, even when AMU on pig farms was comparatively low, pig isolates had increased likelihood of being MDR; harbouring relatively more resistances than those from other livestock species. Therefore, our results indicate that AMR prevalence in livestock is not only influenced by recent AMU on-farm but also livestock-related factors, which can influence the AMR burden in these reservoirs and its plasmid mediated transmission.

## Data Summary

Supplementary Data can be found at https://doi.org/10.6084/m9.figshare.14398127.v1.

**Impact Statement**

The emergence and spread of AMR is of concern with respect to both veterinary and public health, and necessitates investigation of associated factors. This in-depth genomic epidemiological analysis comparing AMR with AMU in three livestock species is, to our knowledge, the first of its’ kind. We identified that use of some antimicrobials was strongly associated with the presence of AMR, strengthening the body of evidence that AMU has an effect on AMR prevalence. Despite low AMU on some pig farms, pig isolates had the highest AMR burden of the three livestock species. Pig isolates were also more likely to harbour AMR plasmids, which are known vehicles of AMR transmission, compared with cattle and sheep isolates. These novel genetic findings demonstrate that differences related to animal host species play an important role in the occurrence of AMR and its possible dissemination through the food chain; informing decisions for AMR control and mitigation and providing directions for future investigations in livestock to assess risk.

## Introduction

Antimicrobial resistance (AMR) is a global health concern, threatening the effective treatment of bacterial infections in both human and veterinary medicine [1, 2]. The rise and spread of AMR has been associated with antimicrobial usage (AMU), providing a selection pressure for multi-drug resistant (MDR) bacteria [3]. National AMU has been significantly correlated with levels of antimicrobial resistant *Escherichia coli* isolated from cattle, pigs and poultry in several European countries [4]. Antimicrobial usage in livestock varies between different livestock sectors; in the UK, the pig sector has a higher AMU (mg kg^−1^) [5, 6] compared with the cattle and poultry sectors, with no evaluable data for the sheep sector. Nevertheless, UK veterinary scanning surveillance of AMR in *E. coli* isolates has indicated a high burden of AMR in cattle and pig isolates, and a lower burden in sheep isolates [6]. Furthermore, molecular characterisation of isolates from livestock, particularly pigs, indicates that livestock-associated *E. coli* may harbour multiple AMR genes including those associated with resistance to important antimicrobials used for human therapeutics including ‘last resort’ antibiotics [7–13]. Bacteria from pigs have been significantly associated with AMR in a metagenomics study of pigs and poultry from nine EU countries [14]. However, few studies have characterised AMR genes present in isolates from healthy sheep, cattle and pigs contemporaneously, making it difficult to assess whether prevalence and types of AMR genes may be influenced by a variety of factors associated with livestock species, as well as antimicrobial usage.

AMR determinants are frequently associated with mobile genetic elements (MGEs) which facilitate horizontal AMR gene acquisition [15], promoting rapid evolution and environmental adaptation. Plasmids from members of the order *Enterobacterales* can be classified into more than 27 incompatibility (Inc) or replicon families [16] with more than half of publicly available plasmid sequences remaining unclassified, indicating that our understanding of plasmid population genetics remains limited [17]. A study by Day *et al*. of *E. coli* from human, animal and food origin in three EU countries, found that the most common AMR plasmids included IncF, IncA/C and IncL/M replicon families, and showed different replicon distributions in human and animal hosts, as well across different sequence types of *E. coli* [18, 19]. Although genome sequencing has facilitated significant insights into plasmid populations present in livestock in recent years [20], the available data on within-family diversity and timeframes of evolutionary adaptation remain limited.

This study aimed to use genomics to characterise the AMR genes found in different livestock species (cattle, pig, and sheep) in a defined geographic region in south-central England, to explore livestock AMR prevalence by host species in addition to any associations with on-farm antimicrobial usage (AMU). We focussed on AMR gene presence in members of four key genera of the order *Enterobacterales* (* Escherichia *, *Klebsiella *, *Citrobacter* and *Enterobacter *), the most common species was *E. coli *. We used short- and long-read sequencing to detect AMR genes and fully resolved chromosome and plasmid sequences, so that the genetic context of acquired AMR genes, and plasmid diversity in the order *Enterobacterales* within different livestock species, could be characterised.

## Methods

### Farm recruitment

Five cattle farms, five sheep farms and four pig farms were recruited within 60 km of each other in a defined area of south-central England, in a medium pig-density [21] and cattle-density area [22], and a low sheep-density area [23]. Animal and Plant Health Agency (APHA) databases were used to identify farms within the study area. The five largest farms for each species were invited to participate, and if these declined, the next largest farm would be invited. All participating farmers provided written consent for farm sampling, which took place between January and April 2017. A questionnaire completed by farmers collected information on farming practices, including: farm characteristics, hygiene and disinfection of houses, biosecurity, and antimicrobial usage in the preceding three months. Two pig farms consented to access to their electronic Medicine Book (eMB) data, recording all veterinary medicinal product use (gross quantities per quarter). Three cattle farms facilitated access to herd management software where all medicinal use was recorded at the individual animal level. Ethical approval was not sought as sampling from faecal deposits on the ground was deemed outside of the Animal (Scientific Procedures) Act 1986. Cattle farms RH06 and RH07 were under the same ownership, but managed separately. There were no recorded movements of livestock between cattle or pig farms involved in the study [data sources eAML2 (pig) and Cattle Tracing System (cattle)], however it was not possible to ascertain the occurrence of sheep movements between farms.

### Sampling and bacterial isolate collection

Each farm was split into five or fewer epidemiological groups, defined as a group of animals expected to share similar characteristics, and similarly managed (e.g. pigs of the same age in the same building). From each farm a total of ten pooled faecal samples were collected, to representatively cover each epidemiological group. The types of animals present on the farm included within the pooled sample are detailed in Table 1. Each single sample was made up of small pinches of fresh faeces from the floor, and combined to form a small, golf ball-sized, composite sample. The ten faecal composite samples were pooled, diluted up to 10^−5^ in phosphate buffer solution (pH7.2) and dilutions plated on to CHROMagar ECC (CHROMagar Microbiology) and CHROMagar ECC plates containing 1 mg l^−1^ cefotaxime. Up to 10 colonies (* E. coli* appear blue and other coliforms appear mauve) were collected from 1 mg l^−1^ cefotaxime-supplemented plates and 14 colonies from CHROMagar ECC plates; however, where less than 10 colonies were recovered from the cefotaxime-supplemented plates, additional colonies were taken from the CHROMagar ECC plates, resulting in 24 isolates per farm. At this stage in the selection process, where possible, isolates were selected on the basis of morphological characteristics (colony colour) to maximise the sampling of bacterial diversity from each farm. Pure isolate sub-cultures (*n*=336) were subsequently stored at −80 °C in MicroBank beads (Pro-Lab Diagnostics), and the bacterial species confirmed using MALDI-TOF (Bruker) or 16S rRNA sequencing [24].

**Table 1.** Farm characteristics and antimicrobial usage

Farm ID	Species	Production type	Housing at the time of sampling	Herd size	Number of different active antimicrobial ingredients used*	Number of antimicrobial classes used*	Total usage of antimicrobials (mg in 1000s)*	Total usage of antimicrobials (mg kg−1)*	Number of isolates with a multi-drug resistant genotype†
**RH01**	Pig	Farrow to Finish	Breeding – indoor Grower and finisher – outdoor	3000+	10	7	160846	1040	11
**RH02**	Pig	Grower to Finisher	Indoor	2001–3000	8	5	6736	363	13
**RH03**	Pig	Farrow to Finish	Outdoor	1001–2000	0	0	0	0	2
**RH04**	Pig	Finisher	Indoor	1001–2000	3	2	57	25	4
**RH06**	Cattle	Dairy	Youngstock – indoor Adult – outdoor	750–1000	6	2	480	284	0
**RH07**	Cattle	Dairy	Youngstock – indoor Adult – outdoor	251–500	10	5	104	79	0
**RH08**	Cattle	Dairy	Indoor	501–750	3	3	35	82	0
**RH09**	Cattle	Dairy	Youngstock – indoor Adult outdoor	501–750	13	8	706	670	2
**RH10**	Cattle	Dairy and Beef Finisher‡	Indoor	751–1000	16	7	45	109	3
**RH11**	Sheep	Lowland	Outdoor	101–500	0	0	0	0	0
**RH12**	Sheep	Hill	Outdoor	501–1000	1	1	2	2	0
**RH13**	Sheep	Lowland	Outdoor with a group indoors	101–500	0	0	0	0	0
**RH14**	Sheep	Lowland	Outdoor	2000+	1	1	6	13	0
**RH15**	Sheep	Lowland	Indoor with a small group outdoors	101–500	0	0	0	0	0

*Used in the 3 month period prior to the farm sampling visit.

†Isolates which harboured AMR genes to three or more different classes of antimicrobials.

‡Only dairy cattle sampled.

### Whole genome sequence analysis

A subset of isolates of members of the order *Enterobacterales* (168/336) were selected for sequencing in order to representatively maximise diversity of the target bacterial species, evenly across the sampled farms. All isolates were sequenced using Illumina HiSeq 4000 (short-read) and either PacBio or Nanopore technology (long-read) as described previously [25, 26] (Table S1). Kraken was used to confirm MALDI-ToF species identification results [27].

Hybrid assemblies were created using Unicycler (‘normal’ mode with a --min_component_size and --min_dead_end_size of 500) [25, 26, 28] to fully reconstruct bacterial genomes, and annotated using PROKKA 1.13.3 [29]. Isolates of members of the genus *Escherichia* were assigned to phylogroups using ClermonTyping (v1.4.1) [30]. *In silico* multilocus sequence typing (MLST) of *E. coli* isolates [31] was performed using either SRTS2 [32] or the Technical University of Denmark (DTU) pipeline (https://cge.cbs.dtu.dk/services/MLST/) [33]. The MLST clonal complexes of each ST was identified from the pubMLST.org [34]. The diversity of STs between and within farm was assessed using Shannon Index t-test [35]. SNIPPY (https://github.com/tseemann/snippy) was used to determine single nucleotide polymorphisms (SNPs) for sequenced *E. coli* isolates with respect to the *E. coli* MG1655 reference genome (NCBI accession number: U00096), using default settings. A recombination free maximum-likelihood tree was inferred from the resulting core alignment of 270680 SNPs using ClonalFrameML [36] and RAxML-NG (https://github.com/amkozlov/raxml-ng), with a general time reversible model of nucleotide substitution allowing for across-site rate heterogeneity (GTR-G), and 100 bootstrap replicates. The tree was visualised and annotated in iTOL [37].

The presence of acquired antimicrobial resistance (AMR) genes (an in-house database of 2122 AMR genes associated with 19 antimicrobial classes) and plasmid replicons [38] were determined from Illumina fastq reads using the APHA SeqFinder pipeline [39, 40], which maps unassembled reads to the AMR gene database. Abricate was used to validate and identify genetic context of AMR and plasmid replicon genes in the hybrid assemblies (https://github.com/tseemann/abricate). AMR genes were considered present if 100 % of the gene was mapped to the reference, with the exception of *aph3-Ia*, *ant3-Ia*, *floR*, *qnrB10*, *InuF* and *sul3* that were present at greater than 82 % gene mapping, and the AMR genes were also identified in the Abricate output, which indicated the chromosomal or plasmid location of the genes as well if multiple copies existed in the isolates. The plasmid finder database (https://cge.cbs.dtu.dk/services/PlasmidFinder/) was downloaded in May 2017, where multiple alleles of a rep-type existed these were given an identifier (Fig. S1, available in the online version of this article). Genotypic resistance to the following antimicrobial classes was determined: extended-spectrum cephalosporins (ESCs), non-ESC β-lactams, tetracycline, aminoglycoside, phenicol, trimethoprim, sulphonamide, macrolide/lincosamide/streptogramin, streptothricin and fluoroquinolones [quinolone resistance region determining mutations (QRDR in *gyrA* and *parC*; i.e. chromosomal) and/or plasmid-mediated quinolone resistance (PMQR)]. Genotypic multi-drug resistance (MDR) was defined as an AMR gene profile conferring resistance to three or more of these antimicrobial classes, intrinsic resistance genes were excluded (Table 2). The chromosomal location of AMR genes was inferred from the Abricate result, we selected a fragment of sequence which included the AMR genes and 5 kb upstream and downstream, and used BLASTn to identify similar sequences in the NCBI database. The top hits showing significant homology were subsequently downloaded as Genbank files. Easyfig 2.2.2 [41] was used to visualise the location of multiple AMR genes integrated into the chromosome of *E. coli* isolates from pig and cattle farms, and to compare them with similar sequences identified in the NCBI database.

**Table 2.** Summary of AMR genes identified on livestock farms. Numbers denote numbers of isolates in each category

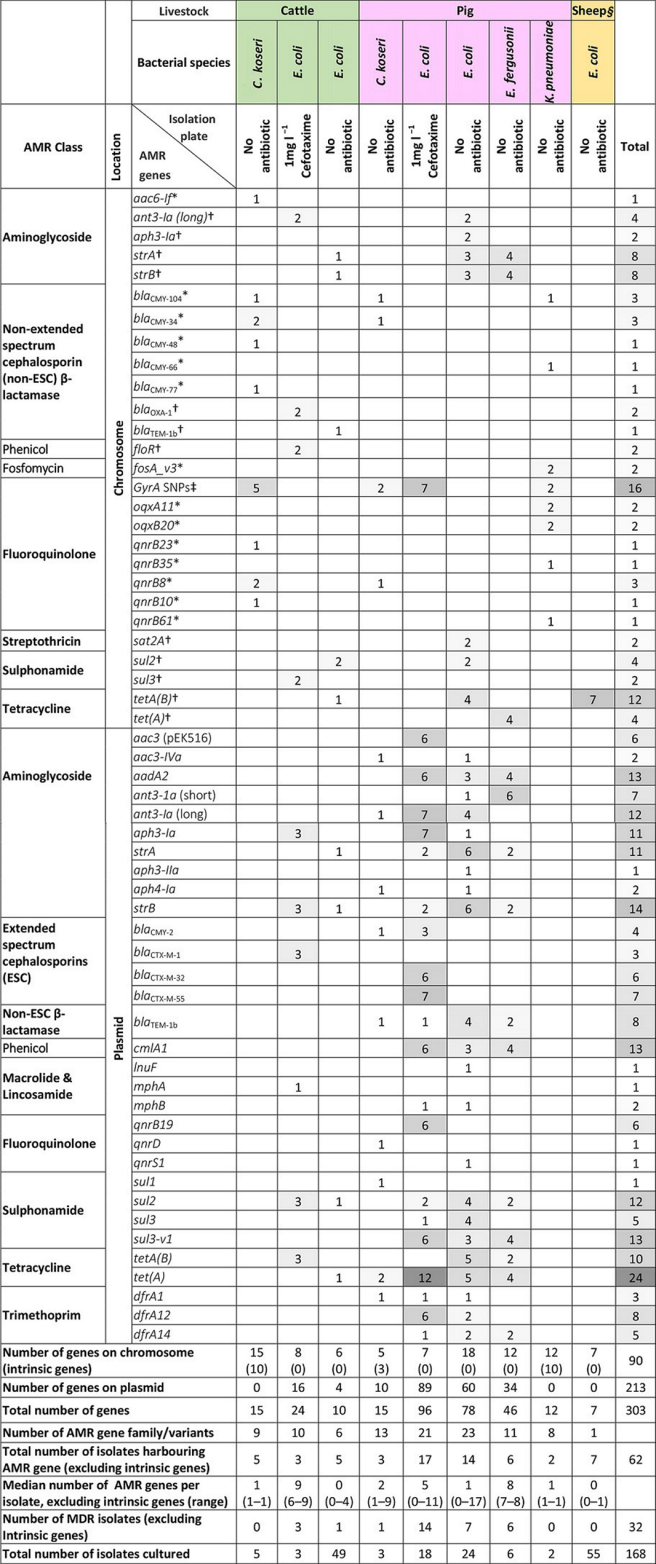

*Intrinsic AMR genes.

†AMR genes frequently associated with plasmids were integrated into *E. coli* and *E. fergusonii* chromosomes.

‡SNPs identified in the quinolone resistance-determining region of *gyrA*.

§An additional three *E. fergusonii* isolates were recovered from one sheep farm (*n*=2) and one cattle farm (*n*=1), which were not included in table as they did not harbour any AMR alleles.

To assess plasmid similarity, k-mer signatures of each reconstructed IncFII, IncI1 and IncX1 plasmid sequence were computed with Sourmash v2.1.0 using the suggested MinHash resolution (1000 : 1 compression ratio) and a k-mer size of 31 [42, 43]. The k-mer signatures were used to compute Jaccard similarity indices (JI; JI=1 means identical plasmids, JI=0 means completely different plasmids). Plasmids were grouped into approximate clusters using MOB cluster in MOB suite, using default settings (distance=0.05) [44]. Roary (3.12.0) was used to define the pangenome of the three common IncFII variants, IncI1 and IncX1 plasmids using default settings [45].

### Statistical analysis

AMR genes were combined to create a single binary variable cataloguing the presence or absence of one or more AMR genes associated with resistance to each antimicrobial class; intrinsic genes were excluded (Table 2). Antimicrobial classes with less than five isolates harbouring resistance genes were excluded, in addition to bacterial species with less than five isolates (species of the genus *Klebsiella *). Isolates selected from CHROMagar ECC containing 1 mg l^−1^ cefotaxime plates were excluded from the analysis of AMU vs AMR due to the increased sensitivity of AMR detection.

Antimicrobial usage (AMU) was summarised as the amount (in mg) of each active ingredient used per farm in the three month period prior to sampling. The National Office of Animal Health (NOAH) Compendium (http://www.noahcompendium.co.uk) was used to determine the mg of each active ingredient per ml of product [46]; this was multiplied by the quantity of product used per farm. To standardise AMU between farms of different herd sizes and animal species, AMU was combined with the number and weight of animals treated to calculate the AMU in mg kg^−1^ animal treated. Several assumptions were made to calculate the mg kg^−1^ of antimicrobial used (Supplementary Methods).

Multivariable analysis using mixed-effects logistic regression models were utilised to examine the association between AMR gene presence for each antimicrobial class with AMU. Due to the number of assumptions involved in calculating AMU kg^−1^, we investigated the use of the associated antimicrobial class, and AMU of all classes, measured in both mg and mg kg^−1^ of animal treated. The farm identity number (Farm ID) was included as a random effect to account for the expected non-independence of the results from multiple isolates from the same farm. The relationship between the presence of MDR isolates and total AMU of all classes (measured in both mg and mg kg^−1^), and the number of antimicrobial classes used was also explored as described above. Simple univariable mixed effects logistic regression with the random effect of farm ID was used to investigate the relationship between the number of antimicrobial classes used and MDR. T tests and Fisher’s exact tests were used to statistically compare sub-populations. A *P*-value of less than 0.05 was considered significant. All statistical analysis was completed using Stata 15 (StataCorp. 2017. Stata Statistical Software: Release 15. StataCorp).

## Results

### Genomic diversity of *Escherichia* isolated from livestock farms in south-central England

Four pig farms, five cattle farms and five sheep farms were recruited in a defined area of south-central England in 2017; farms were of different herd sizes and production types (Table 1). A total of 336 isolates of members of the order *Enterobacterales* of the four study genera were purified from pooled livestock faecal samples on selective (i.e. containing cefotaxime) and non-selective agar plates. A set of 168 isolates were selected based on morphological characteristics, to maximise the sampling of bacterial diversity on farms, and sequenced using both long- and short-read methods [* E. coli* (*n*=149), *E. fergusonii* (*n*=9), *Citrobacter koseri* (*n*=8), and *Klebsiella pneumoniae* (*n*=2); Table S2]. No isolates of members of the genus *Enterobacter* were recovered in this study. Notably only *E. coli* were recovered from cefotaxime plates, representing 14 % (*n*=21 out of 149) of total *E. coli* sequenced; these were from three pig farms (*n*=18 isolates) and one cattle farm (*n*=3).

**Fig. 1.**
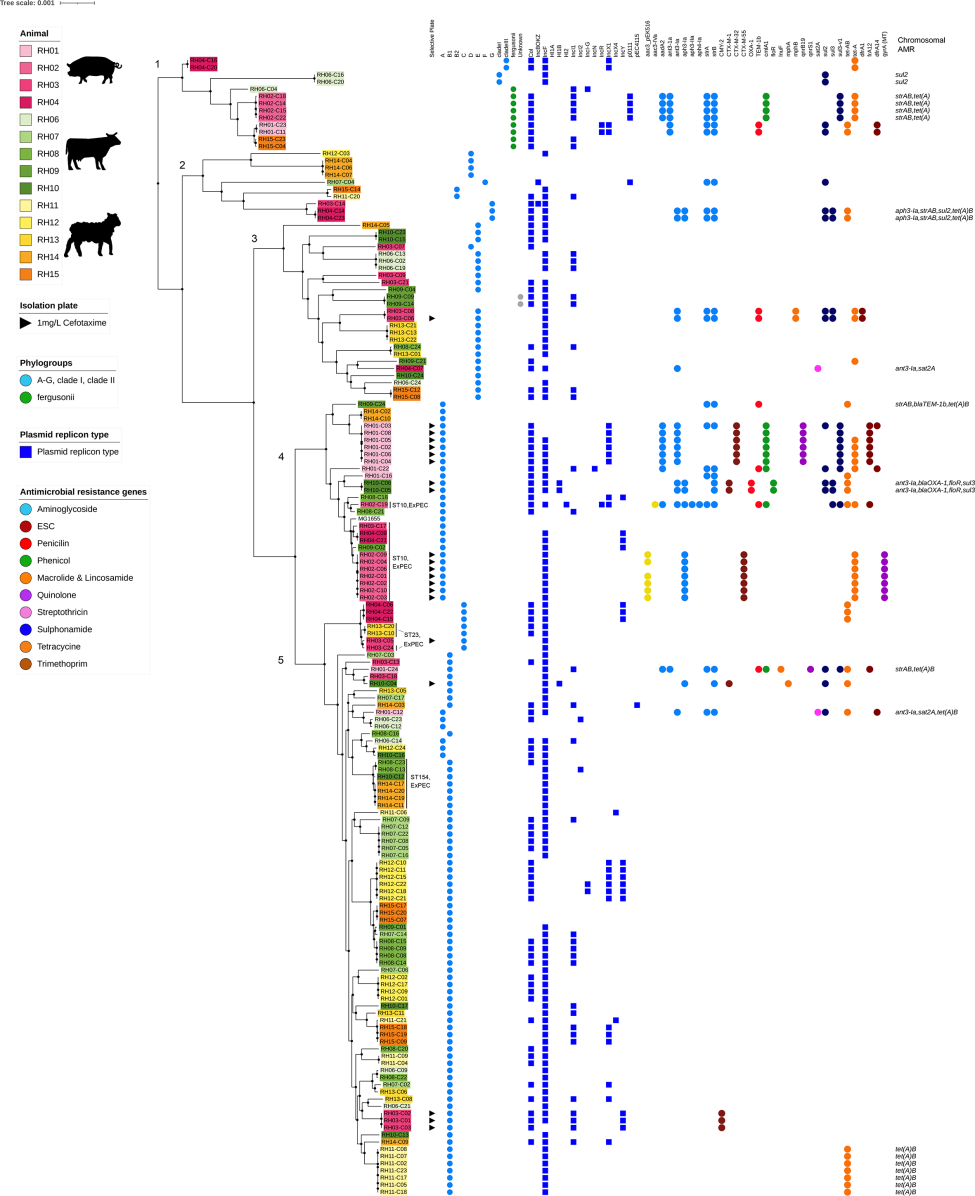
Recombination-free core genome phylogenetic tree reconstruction of *E. coli* and *E. fergusonii *. The isolation plate, phylogroups, plasmid replicons, and AMR genes as well as the chromosomal AMR genes are highlighted for each isolate. The tree is rooted on group A. The isolates' IDs are coloured according to livestock species. Isolates belonging to STs associated with extraintestinal pathogenic *E. coli* (ExPEC) have been highlighted.

Sequenced isolates were diverse, with 72 different sequence types (ST) identified by *in silico* multi-locus sequencing; most were represented only in one host livestock species, but five were detected in multiple host species and included STs associated with extra-intestinal pathogenic *E. coli* ExPEC [47], namely: ST58 (phylogroup B1), ST10 (phylogroup A) and ST23 (phylogroup C) (Fig. 1). *E. coli* from cattle farms had the greatest ST diversity, whilst those from pig farms had the least diversity (*P*=0.006; Fig. S2); but some differences were noted at the farm level irrespective of host species. For example, on pig farm RH02 all *E. coli* recovered were ST10, compared with pig farm RH03 where 11 different STs, from different clonal complexes (the most common locus shared between the isolates was *recA* allele 2 shared by five STs), were recovered.

We restricted strain-based genomic analyses to members of the genus *Escherichia* as only 10 members of other genera were isolated. As expected, *E. fergusonii *, isolated from one sheep, one cattle and two pig farms, formed a separate phylogenetic cluster (group 1), alongside *E. coli* clades I and III (Fig. 1). The *E. coli* isolates formed four phylogenetic groups, and although there was no obvious clustering by host livestock species, *E. coli* isolates from pigs tended to group separately from *E. coli* from sheep, whereas *E. coli* from cattle were interspersed throughout the phylogenetic tree (Fig. 1). The majority of *E. coli* from groups 1 and 4, isolated from pig and cattle farms, harboured AMR genes; in contrast only a minority of isolates from the other groups harboured AMR genes. Although phylogenetically related *E. coli* were detected from different livestock, we found several examples where only a subset of these isolates harboured AMR genes. For example, of the four *E. coli* which clustered in group 5, only two (RH10/cattle and RH01/pig) harboured AMR genes. Also, we noted general congruity between STs and phylogenetic clustering, although there were some differences. For example, for the ten *E. coli* from pig farm RH01, harbouring five different STs (and from different clonal complex), eight isolates clustered within group 4 and the remaining two isolates clustered in group 5, indicating that eight of the ten isolates were genomically more similar than suggested by the differing STs (Table S1).

### Antimicrobial resistance genes and context in isolates of members of the order *Enterobacterales*

The majority (106 out of 168; 63 %) of isolates of members of the order *Enterobacterales* did not harbour AMR genes present within our database. Amongst those with AMR genes (including intrinsic genes, 62 out of 168; 37 %), 23 different AMR gene families, and 49 different alleles were identified in all isolates (Table 2). Genes conferring resistance to colistin or carbapenems were not detected. The AMR genes (*n*=13) found in members of the genera *Citrobacter* and *Klebsiella* were generally intrinsic, with the exception of three isolates of members of the genus *Citrobacter* that contained plasmids harbouring between one and four AMR genes, conferring resistance to different AMR classes (gentamicin, tetracycline, beta-lactam, trimethoprim, sulphonamide and quinolone; data not shown). As expected, with the exception of one isolate, all the *E. coli* isolated on the selective plate harboured AMR genes and the majority were genotypically multi-drug resistant (MDR, defined as presence of AMR genes conferring resistance to three or more antimicrobial classes) (17 out of 21; 81 %; *P*<0.001, Fisher’s exact test). This is consistent with extended spectrum cephalosporin (ESC) resistance being a selective marker for MDR [48]. Isolates recovered from selective plates and any harbouring intrinsic resistance genes were excluded from further AMR and epidemiological analyses.

AMR isolates, from the non-selective plates (*n*=147), originated from eight out of 14 farms including all four pig farms, three out of five cattle and one out of five sheep farms (Table 2, Fig. S3). The greatest proportion of AMR isolates was from pig farms [71 % (25 out of 35)], followed by cattle [18 % (10 out of 55)], and sheep [12 % (7 out of 57)] (Table S2, Fig. S3). Pig isolates harboured a greater number of AMR genes per isolate (mean 3.94, SD 4.34) compared with cattle (mean 0.27, SD 0.72) (*P*<0.001; *t*-test), with only one AMR gene variant found in the seven positive sheep isolates (mean 0.12, SD 0.33).

Of the isolates recovered from the non-selective plates, 10.2 % (15 out of 147) were genotypically multi-drug resistant (MDR, defined as having AMR genes conferring resistance to three or more antimicrobial classes). Those MDR isolates harboured resistance genes to up to eight different antimicrobial classes (Table 2, Fig. S4). It is notable that the highest proportions of MDR members of the order *Enterobacterales* from non-selective plates were from pigs (40.0 %, 14 out of 35) with only 1.8 % (1 out of 55) of cattle and no sheep isolates being MDR (Table S2; *P*<0.001, Fisher’s exact test). A total of nine different AMR gene profiles were identified, each unique to a livestock host; only one MDR profile (but with distinct AMR genes) was shared across two farms (pig farms RH01 and RH02).

Specifically for *E. coli *, isolates from pig farms also had a greater diversity of AMR genes (*n*=23) compared with cattle (*n*=6) and sheep (*n*=1). However, *E. coli* had significantly fewer AMR genes per isolate (mean 0.74, SD 2.38) compared with *E. fergusonii* isolates (mean 5.1, SD 3.63) [*P*<0.001; (*t*-test)]. Notably, 9 out of 23 (39 %) AMR genes found in members of the genus *Escherichia* were chromosomally integrated (Table 2), which were identified in a small number of isolates from the three livestock species. A higher proportion of AMR-containing cattle isolates [60 %, (3 out of 5)] (in addition to two cattle isolates recovered on the selective plates), harboured chromosomal AMR compared with AMR bearing pig isolates [45 %, (9 out of 20)], although this was not statistically significant [*P*=0.323 (Fisher’s exact test)]. The only AMR gene found on sheep farms [*tet(A*)] was chromosomally integrated in all seven ST446 *E. coli* (RH11). We noted that *E. coli* with chromosomally located AMR genes were from three pig farms (RH01, RH02, and RH04), three cattle farms (RH06, RH09, RH10) and one sheep farm (RH11), and were from different STs to those harbouring plasmid-borne AMR either from these or any other farm (Fig. 1).

**Fig. 2.**
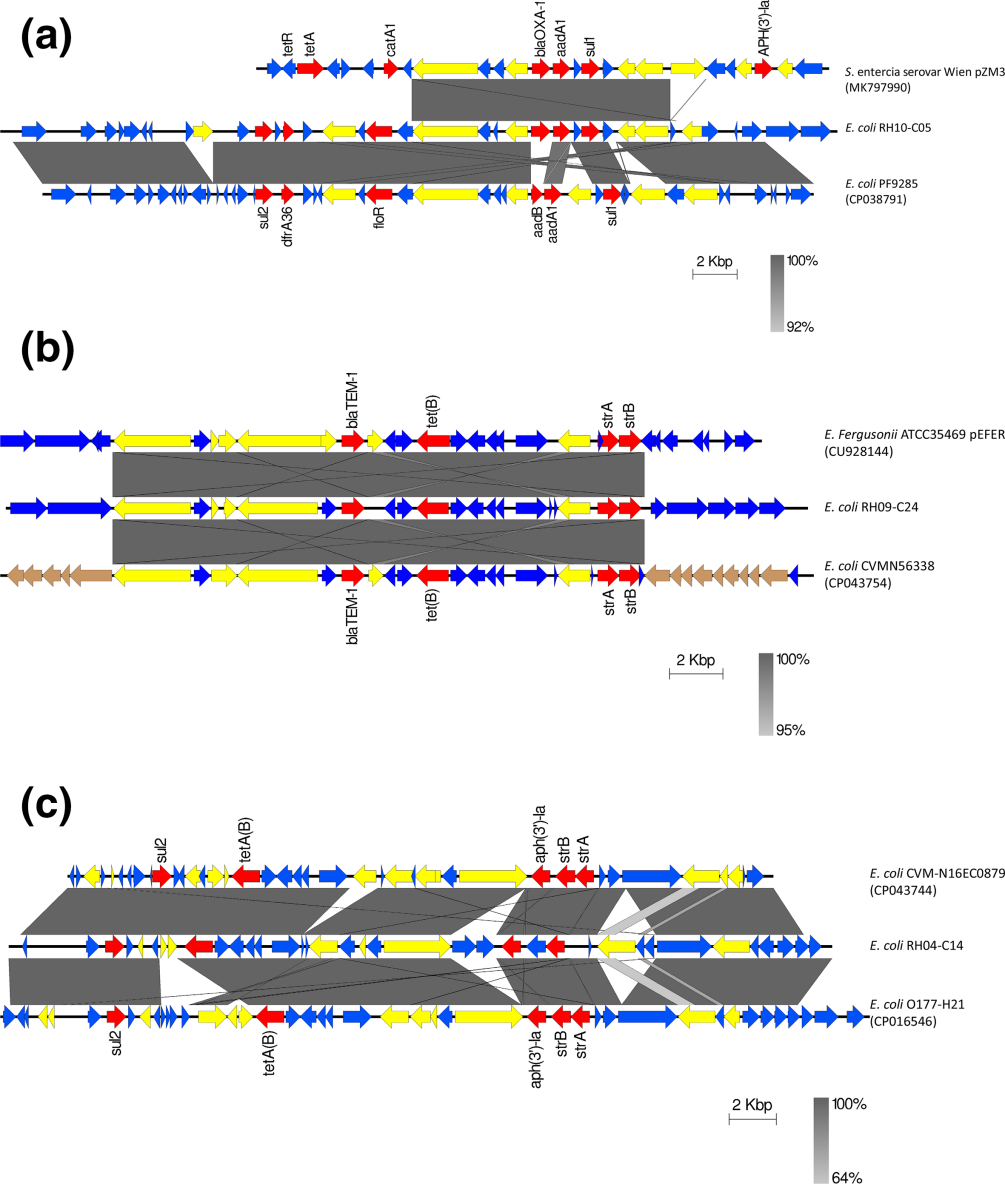
(**a**) Schematic representation of multiple AMR genes integrated into the chromosome of *E. coli* RH10-C05, from a cattle farm. BLASTn comparison of the MDR region integrated into the chromosome of *E. coli* RH10-05 (from cattle farm), plasmid pZM3 from *Salmonella enterica* subsp. *enterica* serovar Wien (Genbank accession number MK797990) (Australia) and the chromosome of *E. coli* PF9285 (Genbank accession number CP038791) isolated from a veal calf in Switzerland [49]. (**b**) Schematic representation of multiple AMR genes integrated into the chromosome of *E. coli* RH09-C24, from a cattle farm. BLASTn comparison of the region harbouring multiple AMR genes in pEFER from *E. fergusonii* ATCC35469 (CU928144) isolated from human faeces (USA), the MDR region integrated into the chromosome of *E. coli* RH09-C24 from a cattle farm (middle line), and the same MDR region in the chromosome of *E. coli* CVM N56338 (CP043754) isolated from pork chops (USA). (**c**) Schematic representation of multiple AMR genes integrated into the chromosome of *E. coli* R04-C14, from a pig farm. BLASTn comparison of the MDR region integrated into the chromosome of *E. coli* RH04-C14 from pig farm, *E. coli* CVM-N16EC0879 (CP043744) isolated from ground turkey (USA), and the chromosome of *E. coli* O177-H21 (CP016546) isolated from human faeces (The Netherlands). The vertical shaded blocks between sequences indicate sequence of shared similarity according to BLASTn. AMR genes are indicated by red arrows, insertion elements transposons and integrases are indicated in yellow, phage associated open reading frames are indicated in brown and the blue arrows are other open reading frames. Figure was created Easyfig 2.2.2.

Six out of 21 isolates with chromosomally integrated AMR harboured resistance to more than three AMR classes. The results of genomic assessment of these six MDR isolates from different STs (pig, ST117; cattle ST206 and ST540), indicated that multiple transposons, insertion sequences and/or phage markers flanked the AMR genes (Fig. 2a–c), reflecting the different mobile elements which may have facilitated the transmission of these genes. Comparison of the MDR cassettes with other sequences from members of the order *Enterobacterales* indicated that these genes have previously been detected chromosomally in other *E. coli* lineages, plasmids and sample types from different continents and timeframes [49, 50] (unpublished data); indicating chromosomal integration of AMR gene cassettes following horizontal gene transfer.

### Antimicrobial usage on livestock farms

Antimicrobial usage (AMU) in the three months prior to sampling varied greatly between livestock species and farms (Table 1). Non-ESC beta-lactams and tetracyclines were the most commonly used antimicrobials, each being utilised on 7 out of 14 (50 %) farms. Sulphonamides were the most heavily used class in terms of quantity, closely followed by trimethoprim (solely used in conjunction with sulphonamides in this study) (Table S3).

Overall, pig farm RH01 reported the highest AMU at more than 20-fold higher than the next highest user, largely attributable to the in-feed use of six antimicrobial products. After weight and herd size adjustment, farm RH01 remained the highest user of antimicrobials (Table 1). On the other hand cattle farm RH09 used comparatively more antimicrobials per kg than pig farm RH02 (670 vs 363 mg kg^−1^ respectively), which had the second highest total AMU. Cattle farms used more diverse antimicrobial classes [mean: five classes per farm; standard deviation (SD): 3.1] than pig (mean: 3.5; SD: 2.5) and sheep farms (mean: 0.4; SD: 0.54) (Table 1). This was partly due to specific products used on cattle farms, some of which contained up to five active ingredients. Sheep farms had very low AMU, with three out of five farms reporting no usage in the study period.

### Association of antimicrobial usage and AMR genes

**Fig. 3.**
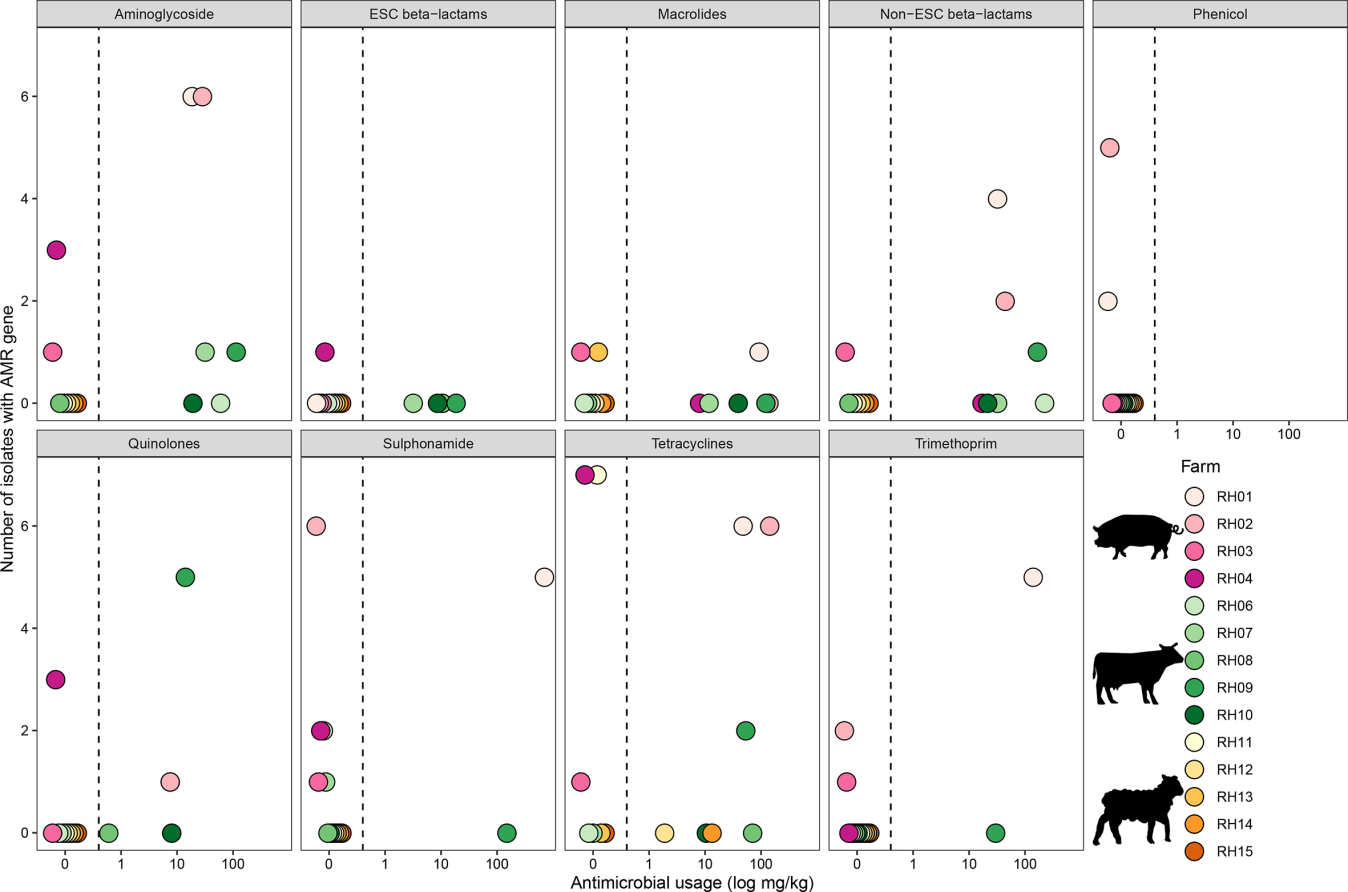
Association of the level of AMU and presence of AMR genes for the 14 different livestock farms. The number of isolates for each farm harbouring AMR genes is indicated on the y-axis and the level of AMU is indicated on the x-axis. Each circle identifies the livestock farm.

To minimise the biases in our analysis of AMU vs AMR, only isolates from non-selective plates and acquired AMR genes were considered (*n*=145); therefore isolates of members of the genus *Klebsiella* (two isolates), isolates purified on cefotaxime supplemented plates (21 isolates), and all intrinsic resistance genes were excluded. Pig farms had a higher proportion of isolates harbouring AMR genes to almost all antimicrobial classes (Fig. 3). Most notably, pig farms RH01 and RH02, the two highest total users of antimicrobials, most consistently harboured the highest numbers of AMR isolates across antimicrobial classes. Interestingly, despite no usage, genes conferring resistance to phenicols were identified in seven isolates from two pig farms (RH01 and RH02). This could be consistent with the co-selection of AMR genes, or the selection and persistence of relevant AMR genes through previous usage. Despite cattle farms also having high AMU during the three month study period, a lower proportion of cattle (18.2 %; 10 out of 55) isolates harboured AMR genes compared with pig farms (69.7 %; 23 out of 33).

The risk of an isolate being MDR increased as the number of different antimicrobial classes used on-farm increased (OR: 2.005; 95 % CI: 0.907–4.434; *P*=0.086), although this result was only approaching significance with this study population. The majority of genotypically MDR isolates (73.3 %; 11 out of 15) were found on pig farms RH01 and RH02, both reporting high AMU. A further three MDR isolates (3 out of 15; 20.0 %) originated from pig farms RH03 and RH04, despite limited AMU reported on these farms. Only one cattle isolate was genotypically MDR (1 out of 15; 6.7 %), originating from farm RH09. This farm reported the highest AMU of the cattle farms, and used eight different antimicrobial classes in the three month study period (Table 1). No isolates from sheep farms were identified as MDR, consistent with their low AMU and the small number of active ingredients used.

A total of 145 isolates of members of the order *Enterobacterales* were included in regression models examining the association between AMU and AMR gene presence. The weight-adjusted use of any antimicrobial class (mg kg^−1^) was significantly associated with the presence of resistance genes to all antimicrobial classes analysed, as well as MDR (Table 3). Weight adjusted AMU of quinolones, tetracyclines and trimethoprim were all positively associated with the presence of an AMR gene encoding respective class-specific resistance (*P*<0.05; Table 3).

**Table 3.** Results of individual multivariable mixed effects logistic regression analysis for presence of each antimicrobial resistance gene for an antimicrobial class and associations with farm usage in mg kg^−1^ of all and corresponding antimicrobial classes. Farm ID was included as a random effect

Antimicrobial class of gene presence	Number of isolates carrying specific class of AMR gene (%)*	Farm usage of corresponding antimicrobial class (mg kg^−1^)			Farm usage of all antimicrobial classes (mg kg^−1^)		
		Odds ratio	95 % Confidence interval	*P* value	Odds ratio	95 % Confidence interval	*P* value
Aminoglycosides	19 (13.1)	1.040	0.962–1.123	0.327	1.010	1.001–1.018	0.021
Non-ESC† beta-lactams	8 (5.5)	1.006	0.979–1.033	0.671	1.005	1.001–1.008	0.002
Phenicols‡	7 (4.8)	–	–	–	1.014	1.001–1.028	0.038
Quinolones	8 (5.5)	1.357	1.167–1.579	<0.001	1.004	1.001–1.007	0.006
Sulphonamides	17 (11.7)	1.001	0.999–1.017	0.101	1.006	1.001–1.011	0.031
Tetracycline	29 (20.0)	1.084	1.018–1.155	0.012	1.011	1.003–1.020	0.010
Trimethoprim	8 (5.5)	1.049	1.009–1.091	0.016	1.007	1.001–1.012	0.013
Multi-drug resistance§	15 (10.3)	–	–	–	1.007	1.001–1.014	0.027

*n=145.

†ESC, Extended spectrum cephalosporins.

‡No on-farm usage of phenicols in the three month period before sampling.

§Isolates were considered multi-drug resistant if they harboured resistance genes to three or more antimicrobial classes.

The relationship between total AMU (i.e. not weight-adjusted) and AMR gene presence was also evaluated (Table S4). The relationship between the use of quinolones and trimethoprim and the presence of corresponding AMR genes remained consistent between the two measures of AMU, with the addition of non-ESC beta-lactam use also being significantly associated with corresponding resistance gene presence. Interestingly, only the presence of genes conferring resistance to non-ESC beta-lactams and trimethoprim remained significantly associated with total AMU of all classes. There was no longer a significant association between AMU and genotypic MDR (*P*>0.05).

### Plasmid population diversity and overlap between species and farm

**Fig. 4.**
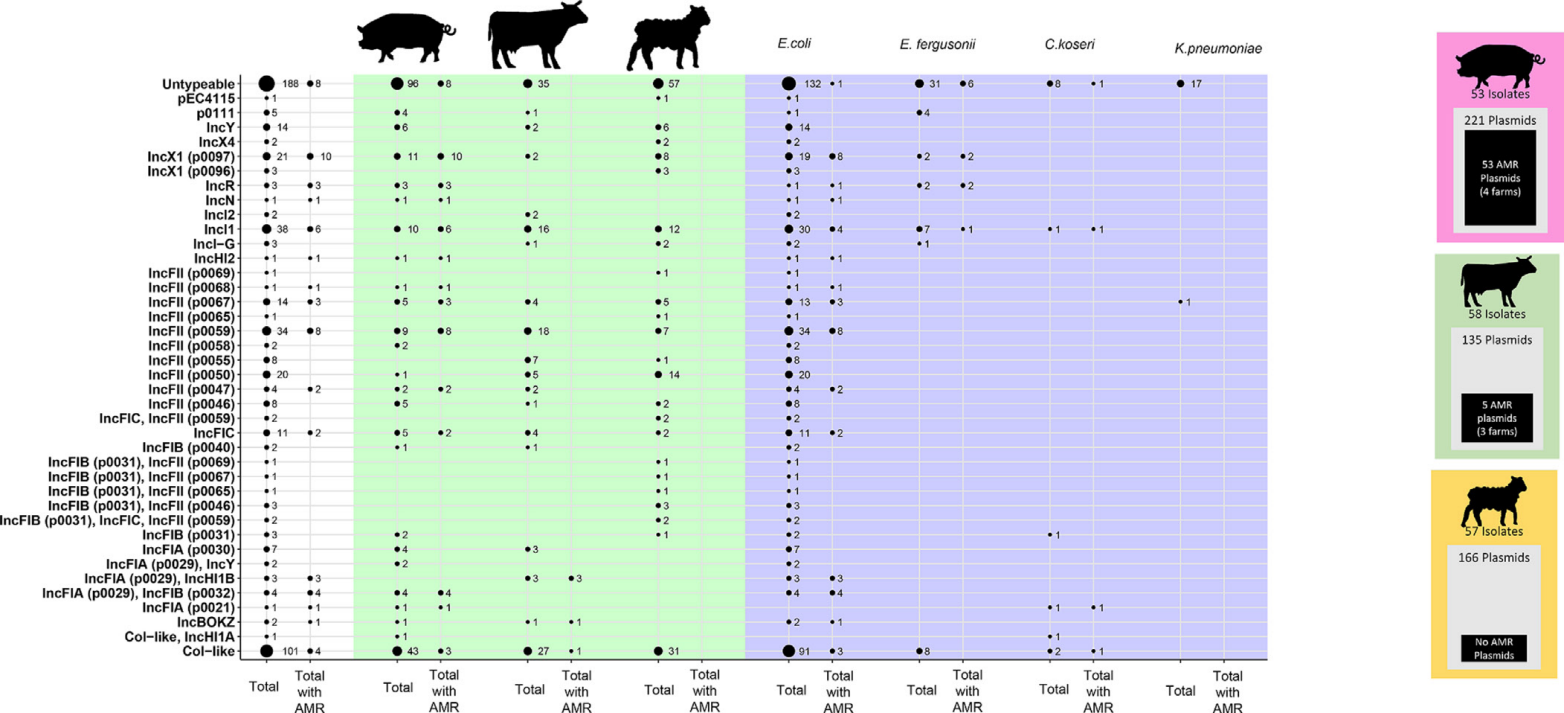
Summary of 522 plasmids found on different livestock farms. Some plasmid types have different alleles, these are represented by (pXXXX). The total number of plasmids for each replicon type as well as those harbouring AMR genes are given per livestock and bacterial species.

The majority (90 %, 152 out of 168) of Enterobacterales isolates recovered from selective and non-selective plates harboured plasmids. In total, 522 plasmids were present in 152 isolates, with some isolates carrying up to 10 plasmids (median: 2, IQR: 1–4 plasmids). Plasmids ranged in size from 1306 to 824863 bp (median: 52614 bp; IQR: 4715–99967 bp). Only two-thirds of plasmids identified harboured a known plasmid replicon (64 %, 334 out of 522), with the most common being col-like replicons [101 out of 522 (19 %)], followed by IncFII [(93 out of 522 (18 %)] (Fig. 4) [25]. Overall 11 % (58 out of 522) of plasmids harboured AMR genes, carrying 1–13 AMR alleles each (median: 4, IQR: 1–4.8) (Fig. S5). Plasmid replicon distributions were similar across all three livestock species, but most of the 58 AMR plasmids identified in our dataset were in *E. coli* from pigs (91 %, 53 out of 58 plasmids), with fewer in cattle (9 %, 5 out of 58), and none in sheep. Additionally, 24 % (53 out of 221) of plasmids detected in pig isolates harboured AMR and of these the majority were genotypically MDR [33 out of 53 (62 %)]. In contrast, only 4 % (5 out of 135) of total plasmids detected in cattle isolates harboured AMR genes, of which three that were MDR had been isolated on selective plates.

**Fig. 5.**
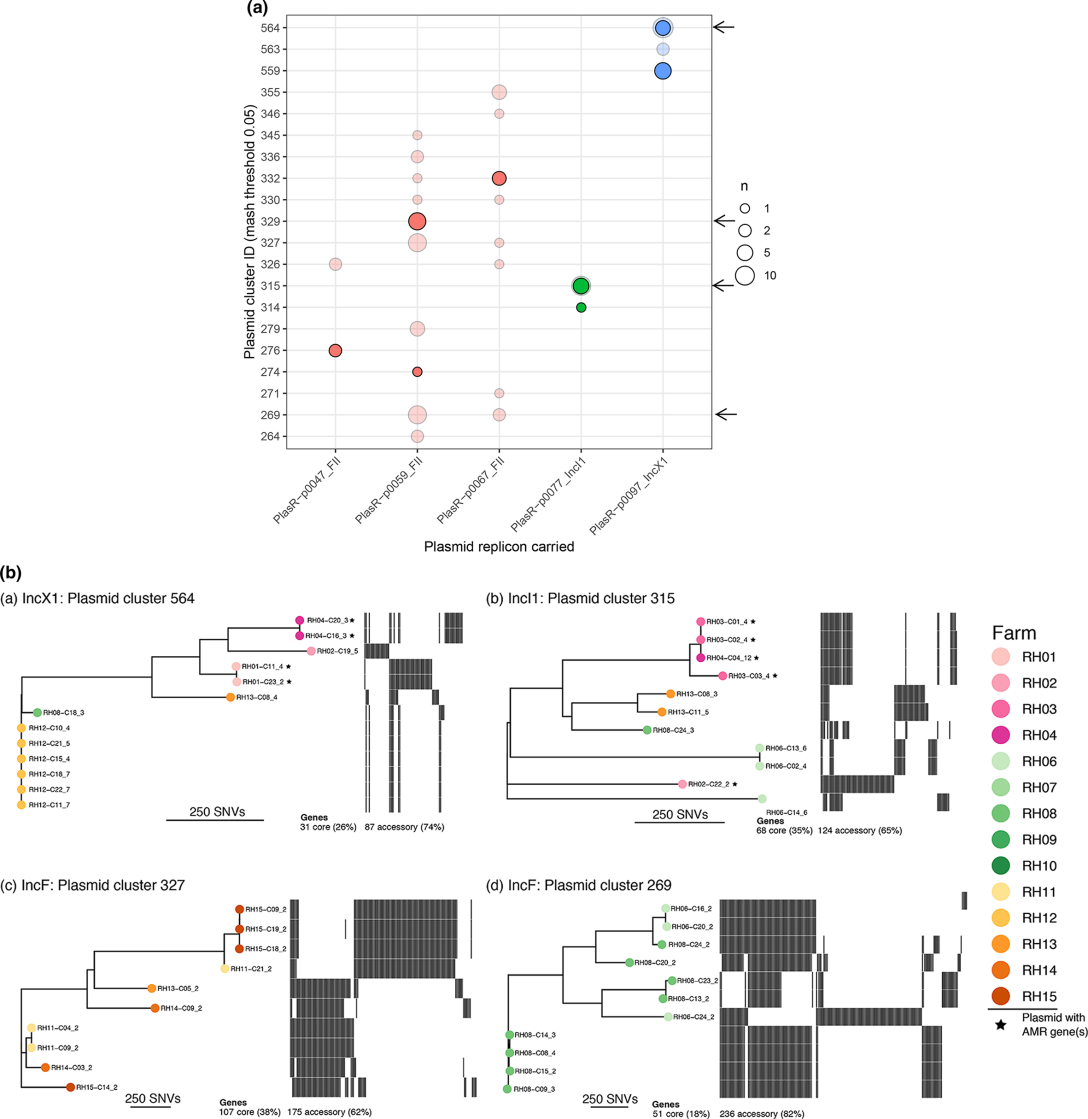
(a) Cluster analysis of the common plasmid replicons. The y-axis shows the plasmid cluster ID based on mob-typing and the x-axis shows the plasmid incompatibility replicon type. Each circle represents the number of plasmids and the darker coloured circles indicate plasmids harbouring AMR genes. The arrows indicate groups further analysed in the centre and right panels. (b) Centre and right panels pangenome analysis of plasmids replicon clusters. Example of the pangenome phylogenies diversity of plasmid sub-clusters. For each plasmid cluster, an alignment of core genes was used to obtain a matrix of pairwise Hamming distances between plasmids (i.e. number of SNVs in core gene alignment). This matrix was then used to reconstruct a neighbour-joining tree. Trees are midpoint-rooted apart from in IncI1 (**b**) where RH06-C14_6 was arbitrarily chosen as the outgroup due to multiple possible roots. The scale bar for each tree shows a distance of 250 SNVs. The number of core genes (used to reconstruct the tree) and accessory genes (shown as a heatmap) are given for each cluster. Phylogeny tip labels, reflecting individual plasmids, are coloured according to farm from which they were isolated. Plasmids harbouring AMR genes are highlighted with a star.

We investigated whether the most common plasmid replicon types (namely IncFII, IncX1 and IncI1) in our dataset were found in multiple livestock species. K-mer analysis of these common plasmid replicon types indicated that although they shared the same replicon type, the remainder of the plasmid genome was dissimilar (Fig. S6a–c); and several plasmid sub-clusters were identified for each replicon type when performing clustering based on mash distances (Fig. 5). Some plasmid sub-clusters were found across livestock species and farms (IncX1 cluster 564, IncI1 cluster 315), whereas some appeared to be restricted to particular livestock species but distributed across farms (IncFII clusters 327 and 269) (Fig. 5). Within each plasmid sub-cluster, there was a diversity of accessory genes. Plasmid phylogenies, reconstructed from the core genes of each plasmid sub-cluster, illustrated the variability of accessory genes within each sub-cluster, although typically some congruence with the core genome phylogeny was observed (Fig. 5b). Plasmids that were closely related with respect to core and accessory genomes were largely restricted to single farms.

For the three most common replicon types present in AMR plasmids from pig isolates, pangenome analysis demonstrated greater diversity in the plasmid genomes within accessory genes found in IncX1 and three IncFII plasmid variants compared with IncI1 plasmids, which had the highest number of conserved core genes, whether they were AMR harbouring or not (Table 4). Compared to the IncFII and IncX1 plasmids, which were very diverse, the IncI1 AMR plasmids shared a greater proportion of their core plasmid genome with non-AMR harbouring IncI1 plasmids. Also a higher proportion of genes were unique to AMR plasmids compared with non-AMR plasmids for IncI1 and a sub-type of IncFII plasmids (defined as p0059 variants in this study); although the reverse was true of IncX1 and the IncFII p0067 variant. Interestingly, the IncX1 AMR plasmids had the lowest number of core genes as well as the highest number of accessory genes, indicating this group of plasmids to be the most diverse of those examined (Table 4).

**Table 4.** Summary of pangenome, core and accessory genes of common plasmid replicon types, with and without AMR genes. The Genbank accession number of plasmid rep-type is given in italics

	IncI1 plasmids			IncFII (p0059) plasmids (AP009242)			IncFII (p0047) plasmids (CP000648)			IncFII (p0067) plasmids (AY458016)			IncX1 (p0097) plasmids (CP003417)		
Plasmid replicon	Total	No AMR	AMR	Total	No AMR	AMR	Total	No AMR	AMR	Total	No AMR	AMR	Total	No AMR	AMR
**Number of plasmids**	12	6	6	35	27	8	4	2	2	11	8	3	21	11	10
**Size range (bp**)		84 336–99 090	90 767–116 881		67 718–195 368	113 309–184 904		153 833–153 833	187 580–187 622		74 290–160 376	80 510–80 510		32 364–45 556	44 007–52 614
**Average number of genes (range**)	105.1	96.8 (91–107)	113.3 (104–135)	144.7	141.4 (131–202)	145.7 (75–210)	180.5	153	208	120.5	130.4 (85–175)	94	50.6	43.4 (41–53)	58.6 (56–69)
**Pangenome**	213	144	174	679	543	252	306	153	208	505	477	94	170	80	135
**Core genes**	32.4% (69)	47.9% (69)	51.1% (89)	4.1 % (28)	6.1% (33)	32.9 % (83)	18% (55)	100% (153)	100% (208)	2% (10)	2.3% (11)	100% (94)	2.4 % (4)	38.8% (31)	4.4% (6)
Accessory proteins *	35.7 % (76)	35.4% (51)	12.1% (21)	67.6 % (459)	70% (380)	20.6 % (52)	82% (251)	0% (0)	0% (0)	46.1% (233)	42.1% (201)	0% (0)	81.8 % (139)	25% (20)	95.6% (129)
**Unique accessory (only one plasmid**)	31.9 % (68)	16.7 % (24)	36.8 % (64)	28.3 % (192)	23.9 % (130)	46.4 % (117)	0 % (0)	0 % (0)	0 % (0)	51.9 % (262)	55.6 % (265)	0 % (0)	15.9 % (27)	36.3 % (29)	0 % (0)

*Genes found in more than one plasmid and less than the maximum number of plasmids.

## Discussion

The aim of this study was to use genomic epidemiology to assess any influence livestock species and AMU may have on AMR genes present in key genera of the order *Enterobacterales* collected from food animals (cattle, pigs and sheep) in south-central England. The main species recovered on the non-selective and cefotaxime selective plates was *E. coli *, and it harboured the majority of acquired AMR genes identified. Furthermore, on-farm AMU of specific classes, in addition to the host livestock species, was closely associated with the presence of AMR genes. Pig farms had the highest risk of harbouring AMR isolates, many of which were MDR. In fact, 40 % of pig isolates from non-selective plates, representing the *E. coli* flora, were genotypically MDR, similar to numbers reported previously [3, 51]. This was substantially higher than the proportion of MDR isolates from cattle and sheep purified on non-selective plates (1.8 and 0 %, respectively).

The majority of acquired AMR genes found in isolates from the non-selective plates, which included ten antimicrobial classes, were identified in *E. coli* from pig farms (nine AMR classes), compared with only one AMR gene (tetracycline resistance) found on one sheep farm. Although pig isolates harboured a greater number of AMR genes per isolate this was due to the identification of MDR *E. fergusonii* isolates, which were not found in the other livestock hosts in this study. In our reconstructed genomes, we identified chromosomal integration of AMR genes in diverse members of the genus *Escherichia.* Although the numbers were small and the difference non-significant in this context, cattle isolates were three times more likely to harbour chromosomal AMR genes than pig and sheep isolates (*P*=0.323). Furthermore, several isolates harboured different chromosomal MDR gene cassettes, which have been identified in *E. coli* as well as plasmids from different countries and compartments including human, food stuffs, and livestock [49, 50, 52–54], highlighting their global distribution.

Phylogenetic analysis highlighted the diversity of *E. coli* present, although there was some genomic similarity, particularly within farms. Within livestock, *E. coli* from pig farms were less diverse than those from cattle and sheep farms. Interestingly from our reconstruction of genomes we showed that although isolates may harbour a plethora of plasmids, only a minority of plasmids (11 %) harboured AMR genes, and these were predominantly present in pig isolates. Pangenome analysis of plasmids from the three most common replicon types demonstrated significant diversity in genomes of plasmids of the same replicon type. Indeed, different genetic content was observed within plasmid clusters, with some clusters containing both AMR and non-AMR plasmids, with most distributed across farms, but some showing a much more restricted niche. The diversity of plasmids present in this dataset has been investigated further elsewhere [25, 55].

The high AMU in pig farms in comparison to cattle and sheep farms in this study mirrors UK national usage data reported in the UK in 2017, where the pig sector reported 20-fold higher AMU than the dairy sector [5]. Nonetheless, reported AMU on pig and cattle farms in this study was, on average, far higher than that seen nationally (357 vs 131 mg kg^−1^ for pig and 245 vs 17 mg kg^−1^ for cattle) [5]. AMU in sheep farms was not covered in the UK national usage report; in this study the average total AMU in sheep was significantly lower than those in cattle and pigs, at only 5.2 mg kg^−1^.

Both total and class-specific effects of AMU on the presence of AMR genes were observed, highlighting the complex interactions between selection pressures and the presence of AMR. The positive association between total AMU (mg kg^−1^) and the presence of AMR genes for all AM classes indicated that increasing AMU, regardless of class, appears to drive AMR emergence to these classes, which is consistent with established concepts of co-selection. By creating a corrected AMU variable which includes the fractional use of other antimicrobials, previous studies have attempted to account for co-selection when examining the relationship between AMU and AMR [56]. This technique has been successfully adopted to demonstrate more complex AMU vs AMR relationships [57], and although not utilised in this study, could provide scope for future analysis.

In contrast to total use, only the class-specific use (mg kg^−1^) of quinolones, tetracyclines, and trimethoprim were associated with the presence of these types of AMR genes, suggesting the possibility of a direct relationship between their use and AMR gene presence. The two sheep farms that recorded AMU within the study period only recorded the use of tetracycline; in these two farms only the presence of *tetA(B*) harbouring *E. coli* was detected. Our class-specific results for sulphonamides and trimethoprim should be interpreted cautiously, as only two farms reported their use in the study period (RH01 and RH09).

We observed some differences in the relationships between AMR and the two different measures of AMU (mg kg^−1^ vs usage in grams). This may reflect the fact that the antibiotic concentrations resulting from administration exerts selection pressures on the colonising members of the order *Enterobacterales* within a herd/flock, and measures accounting for animal biomass may be more representative. One finisher pig farm (RH04), despite its low levels of AMU, harboured high levels of AMR isolates. There may be multiple reasons for this: different/high AMU in the supplying breeding farm; the relatively short AMU study period, which may not capture all relevant AMU history; or other factors not measured which may co-select for AMR e.g. heavy metal usage/concentrations.

We are aware that there are a number of limitations to this study, such as a single defined study area with a limited number of farms; different lifespans of the three livestock species studied which may affect the persistence of AMR; and the use of assumptions in the calculation of AMU, as described in the methods. In addition, the non-random structured selection of isolates from both selective and non-selective media, and the inclusion of only four bacterial species, may have introduced bias in the limited analyses which used isolates from both these plates. However, few studies to date have used fully reconstructed genomes of multiple bacterial species to investigate AMR in different livestock, and correlated it with on-farm factors, such as AMU, to understand prevalence in livestock.

Future work can help address some of the limitations identified here. For example, future studies could explore the relationship between AMR and AMU over time, and investigate whether changes in AMU are associated with the presence and persistence of AMR genes in livestock. Also given the closed genomes this study has generated, identifying plasmids with and without AMR genes may allow us to identify any factors which enable 11 % of plasmids to harbour AMR genes whilst 89 % do not. Also, understanding why the majority of AMR plasmids found in *E. coli* were from pig isolates compared with cattle isolates, even though both farming practices used high levels of AMU. Additionally we identified several examples of chromosomal AMR genes as well as MDR cassettes. In future, investigation of the process of insertion and the possible origin of these genes could be important to understanding how AMR *E. coli* may persist in livestock populations, as if they are stably integrated within the chromosome, the *E. coli* may not be lost when AMU is reduced or stopped on farm. Finally as we examined a small number of farms in a UK region, it would be interesting to see if these observations are replicated in a wider panel of farms.

In summary, *E. coli* was most the prevalent species of the order *Enterobacterales* isolated from livestock, and the most likely to harbour transmissible AMR. The burden of AMR isolates was highest on average in pigs, and these isolates were also more likely to be MDR. This is in contrast to cattle farms which also had high AMU but had lower AMR gene and MDR isolate prevalence, possibly due to differences in the distribution of AMR bearing plasmids. This indicates that factors in addition to those examined in this study, e.g. the livestock species and the farm environment, may play a role in the presence of AMR isolates in livestock; this requires further investigation and may influence future policy control measures for AMR in the food chain.

### Supplementary methods

Standardised weight classifications for each species and age group were used, as per the European Surveillance of Veterinary Antimicrobial Consumption (ESVAC) report [58]. Where a dosage was not specified (7 %; 5 out of 68 records), expert veterinary opinion was sought to estimate the typical expected dosage of antibiotic for the treatment reason and treated animals specified. Where the number of animals treated was not specified, but the total quantity of product used was known, the number of animals treated was calculated using the middle dosage range specified in the NOAH compendium for that product. If the age group of animal was not specified (24 %; 16 out of 68), the reason for treatment, administration route and quantity of product given was used to infer the age group in which the product was used. If the product concentration was not specified (12 %; 8 out of 68), the median concentration of all products available was used. If two types of age group were specified for treatment (10 %; 7 out of 68), the older group was used to provide an average weight of the animal. If the number of animals treated was not provided (24 %; 16 out of 68), but the quantity of product used in the three month period was, it was assumed that an animal received one injection or tube of product. Only antibiotics were included in analysis; information on anthelmintic use was not considered.

## Supplementary Data

Supplementary material 1

Supplementary material 2

Supplementary material 3
