# Supplementary material for: A genomic epidemiological study shows that prevalence of antimicrobial resistance in Enterobacterales is associated with the livestock host, as well as antimicrobial usage
Source: Microb Genom. 2021 Oct 5;7(10):000630. doi: 10.1099/mgen.0.000630 (PMC8627209; doi:10.1099/mgen.0.000630)
Supplement: Supplementary material 1 [file mgen-7-0630-s001.pdf]

Fig. S1 Phylogenetic relationship between plasmid incompatibility replicon types

Plasmids were typed according to the Carattoli *et. al.* plasmid replicon sequences, this figure shows the phylogenetic relationship between the different sequences. Due to the diversity, the IncFII, IncFIA, IncFIB and IncFIC are highlighted. The accession number of each replicon is given in brackets.

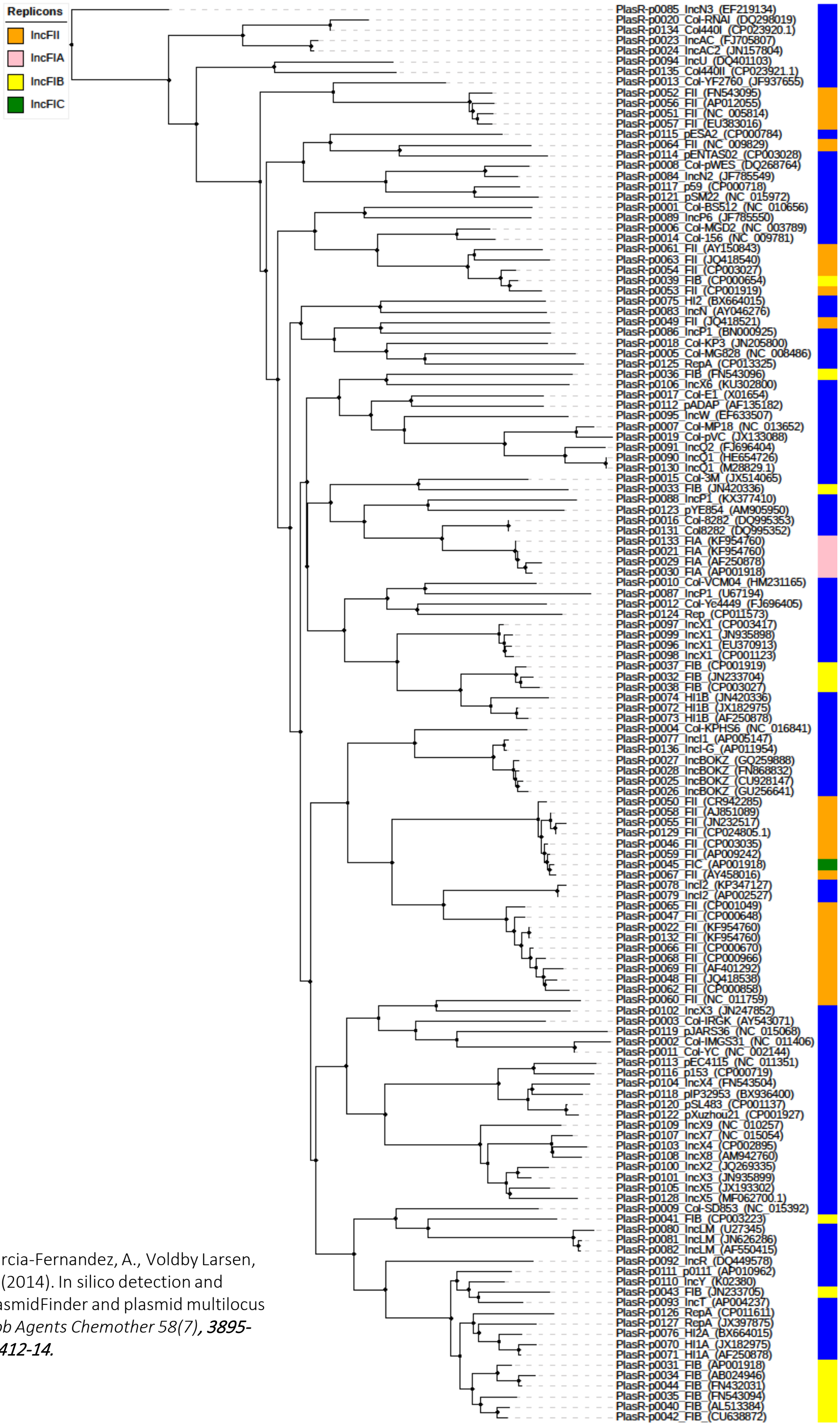

Carattoli, A., Zankari, E., Garcia-Fernandez, A., Voldby Larsen, M., Lund, O., Villa, L., et al. (2014). In silico detection and typing of plasmids using PlasmidFinder and plasmid multilocus sequence typing. *Antimicrob Agents Chemother 58(7), 3895-3903. doi: 10.1128/AAC.02412-14.*

Fig. S2 Diversity of *E. coli* MLST sequence types

The diversity of *E. coli* sequence types identified in dataset. (A) Diversity of each livestock species. (B) Diversity found on each farm. The number of isolates and sequence types identified in each livestock or farm are included in the table.

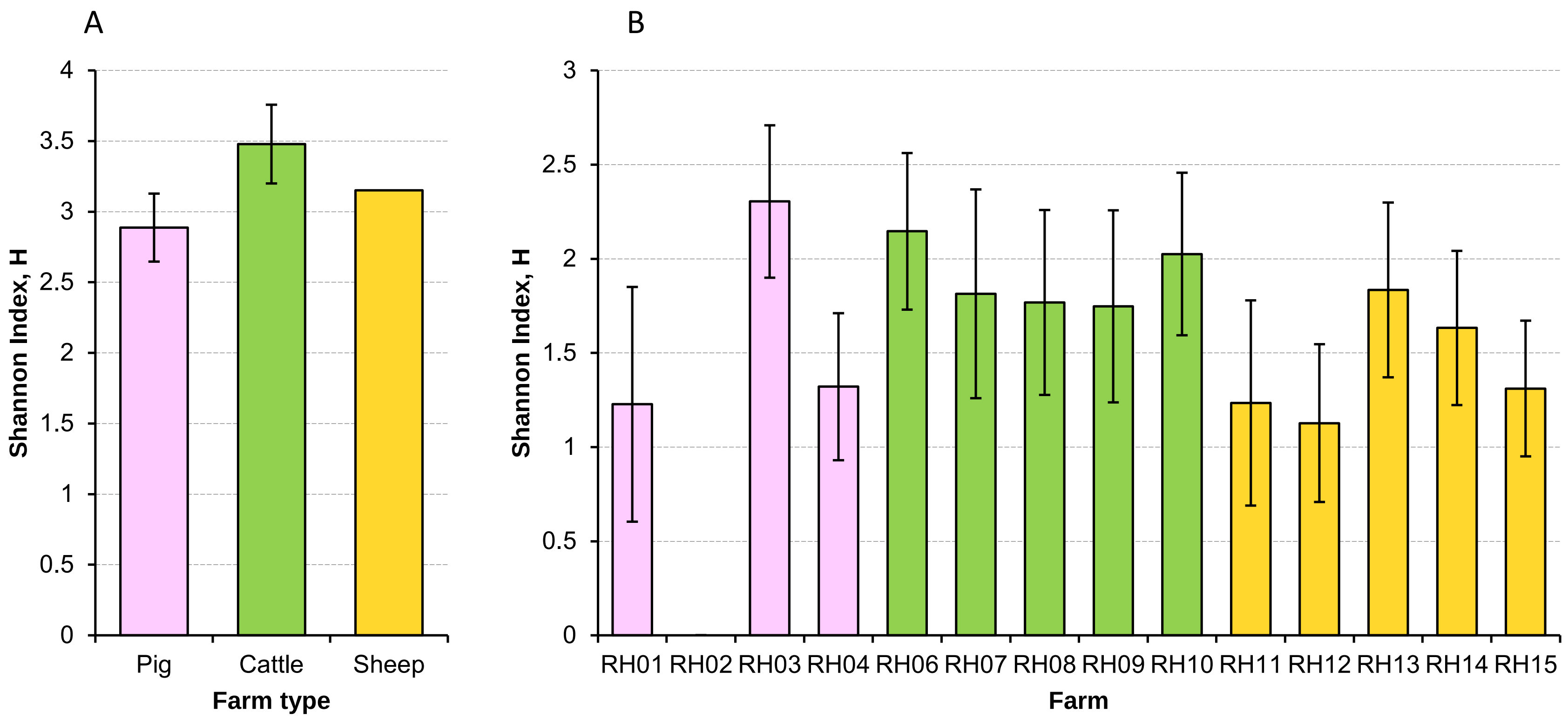

52	42	55	No of *E.coli*	10	8	14	10	11	12	12	7	10	12	12	10	12	9
35	20	24	No of ST	5	1	11	5	9	8	8	6	8	5	4	7	6	4

Fig. S3 Correlation of AMR genes harboured by isolate from cattle, pig and sheep farms, in South Central England.
The number of AMR genes detected by WGS in 168 isolates from fourteen farms. The coloured bars indicate the number of isolates from cattle farms (green), pig farms (pink) and sheep farms (yellow). The hashed bars indicate isolates recovered from the cefotaxime selection plate.

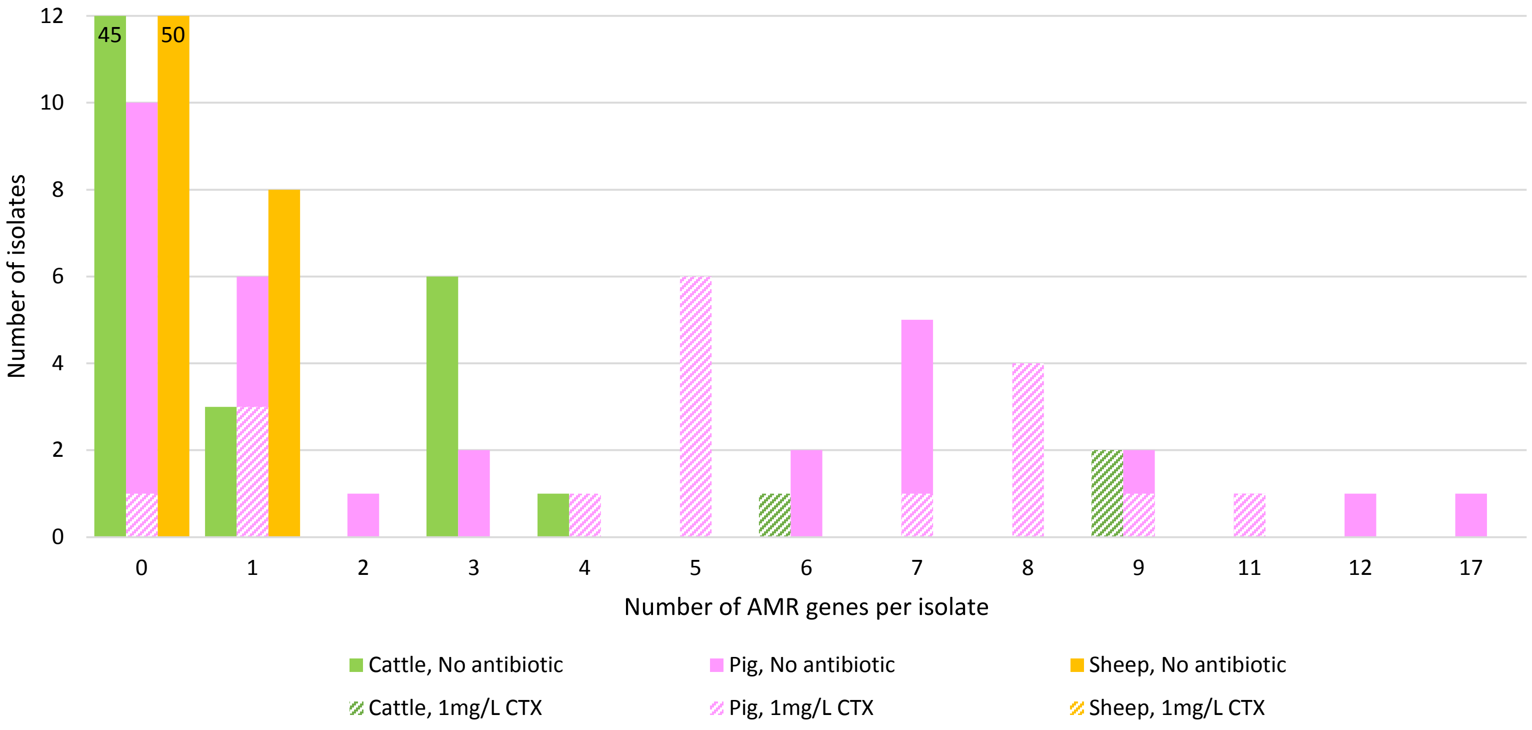

Fig. S4 Correlation of MDR isolates from cattle, pig and sheep farms, in South Central England.
The number of AMR classes detected by WGS in 168 isolates from fourteen farms. The coloured bars indicate the number of isolates from cattle farms (green), pig farms (pink) and sheep farms (yellow). Hashed bar indicate isolates were recovered on cefotaxime selective plates.

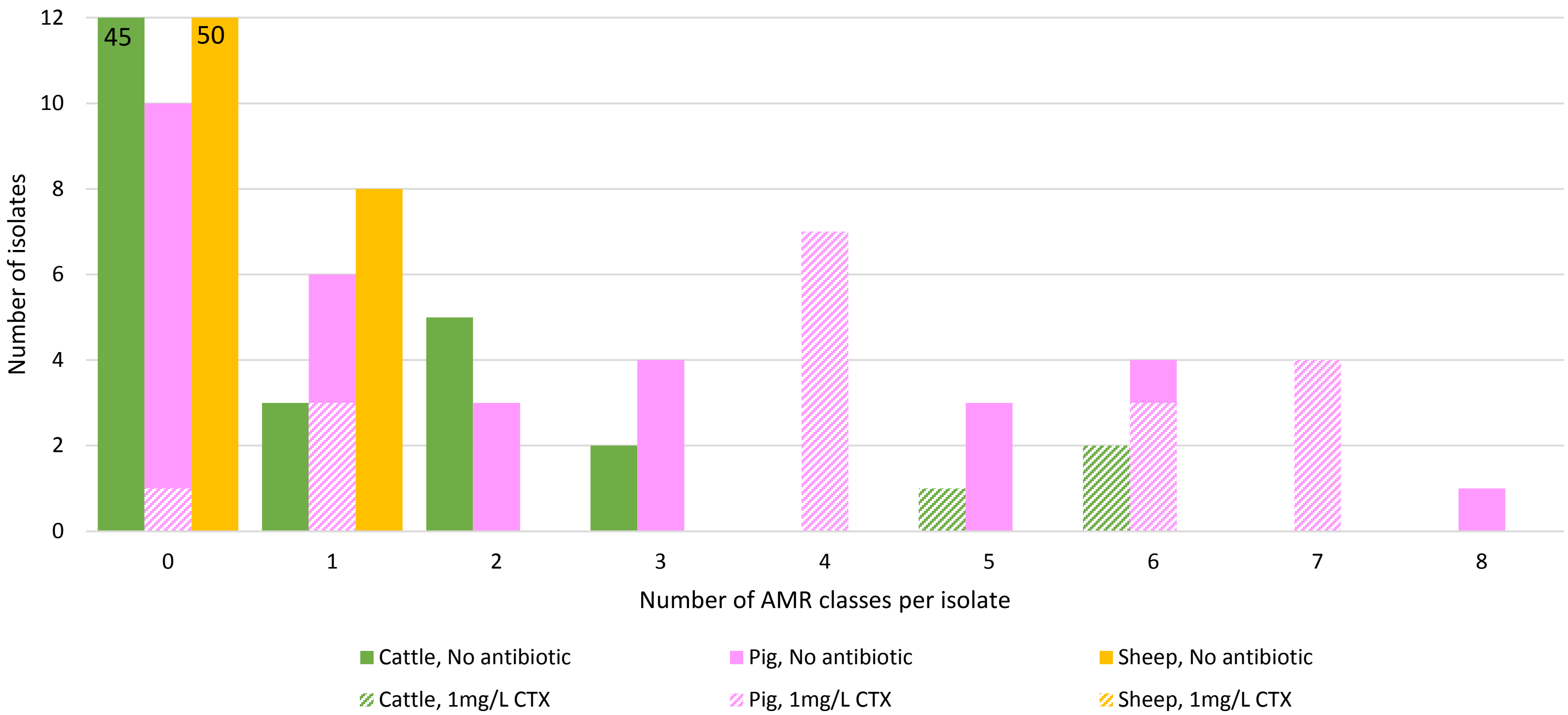

Fig. S5 Number of AMR alleles harboured by plasmid replicon types
Bar chart of the number of different plasmid rep-type harbouring AMR alleles. The numbers in green boxes are the number of isolates from cattle farms harbouring AMR plasmid.

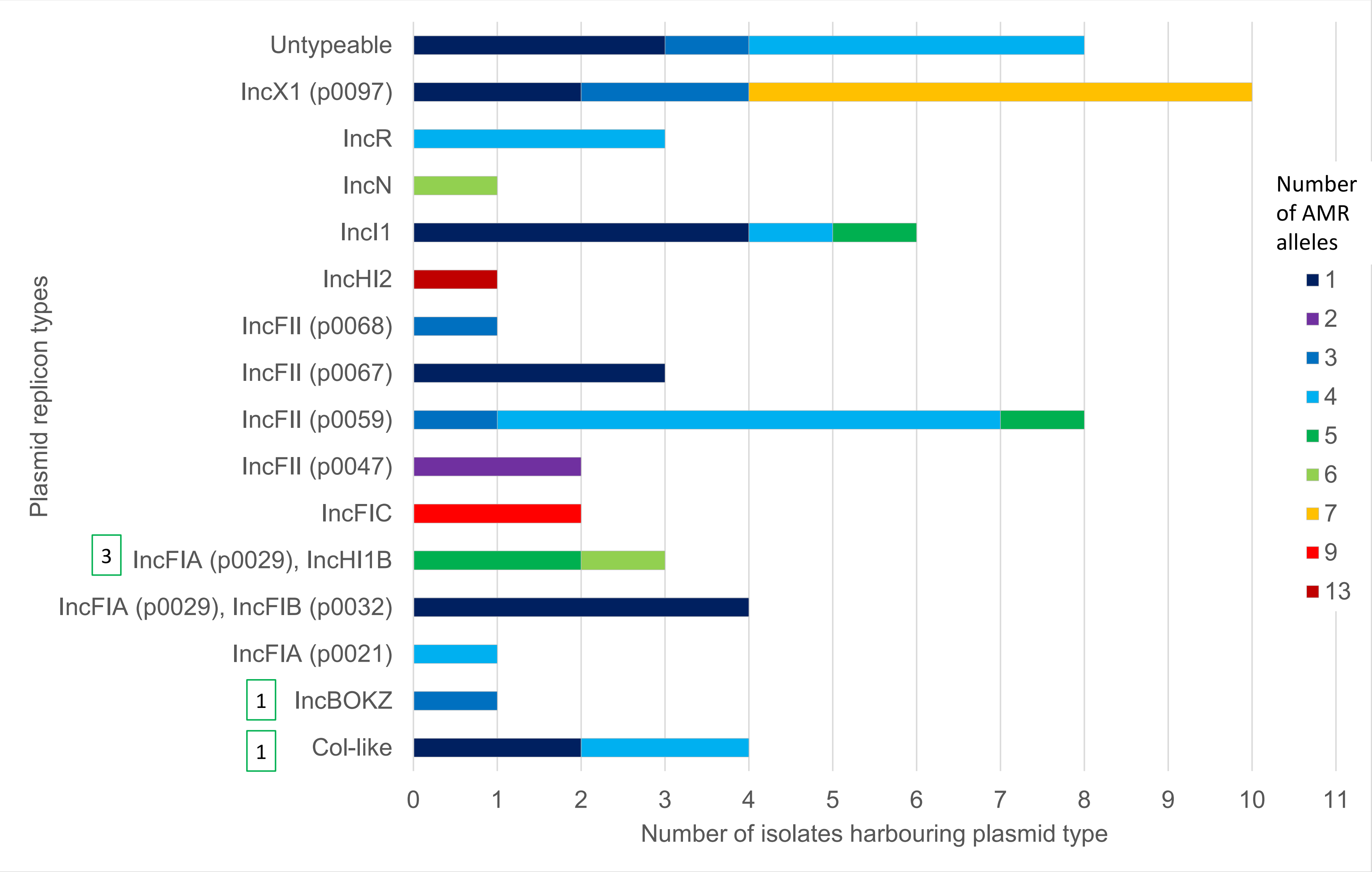

S6 Fig. (A) IncFII, (B) IncX1 and (C) IncI1 - Heat map and dendrogram demonstrating the heterogeneity of the three most common plasmid replicon types, with and without AMR genes. k-mer signatures of each plasmid type were compared using sourmash. The heat map displays the pairwise Jaccard similarity indices between plasmids; the scale is between 0 (yellow) and 1 (blue), where 1 indicated high genome similarity of two plasmids. Plasmids harbouring the same AMR genes are highlighted in different colours. Each plasmids label includes the rep-type, AMR alleles found on plasmid (if present), livestock host, farm ID and bacterial host.

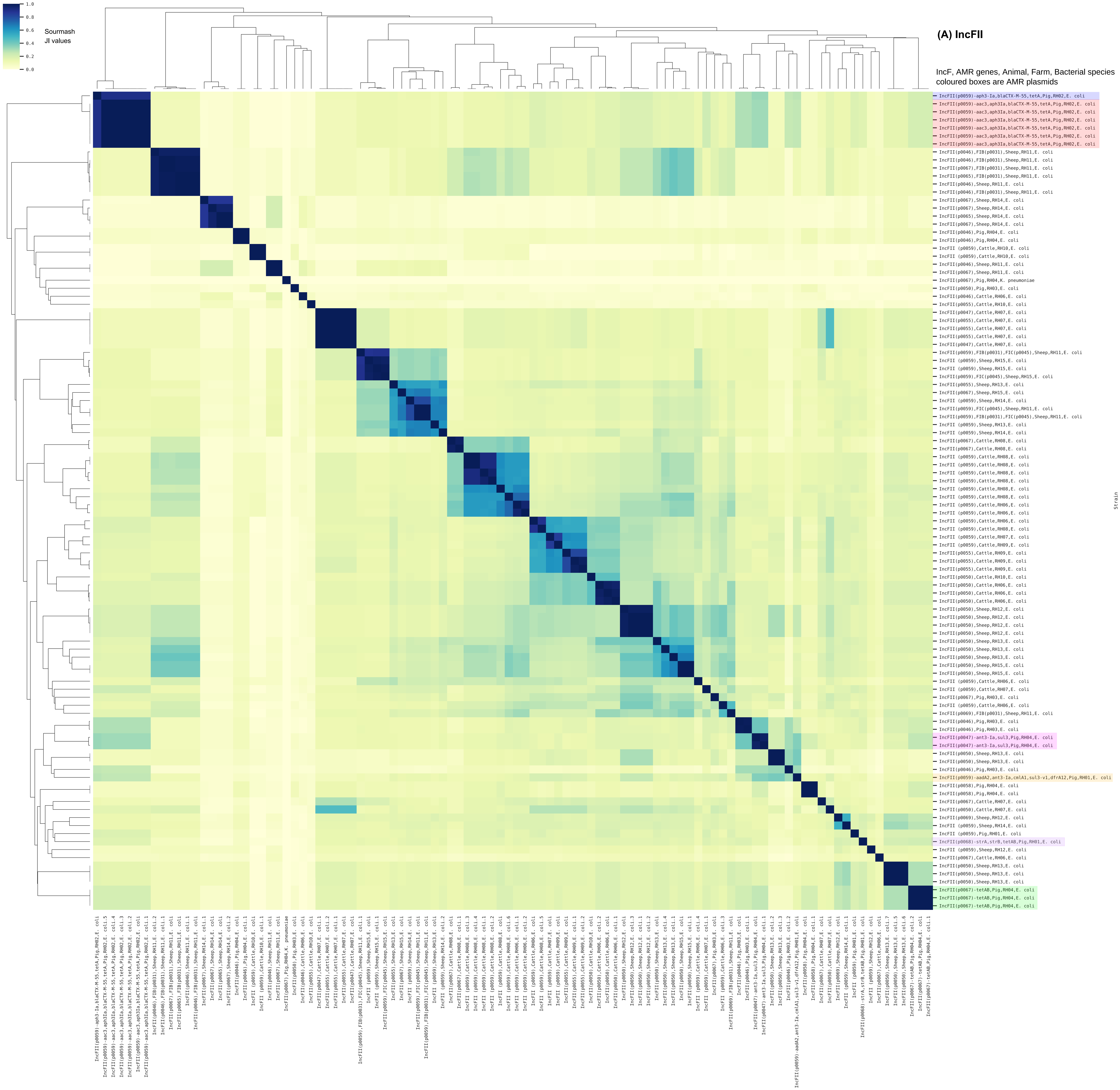

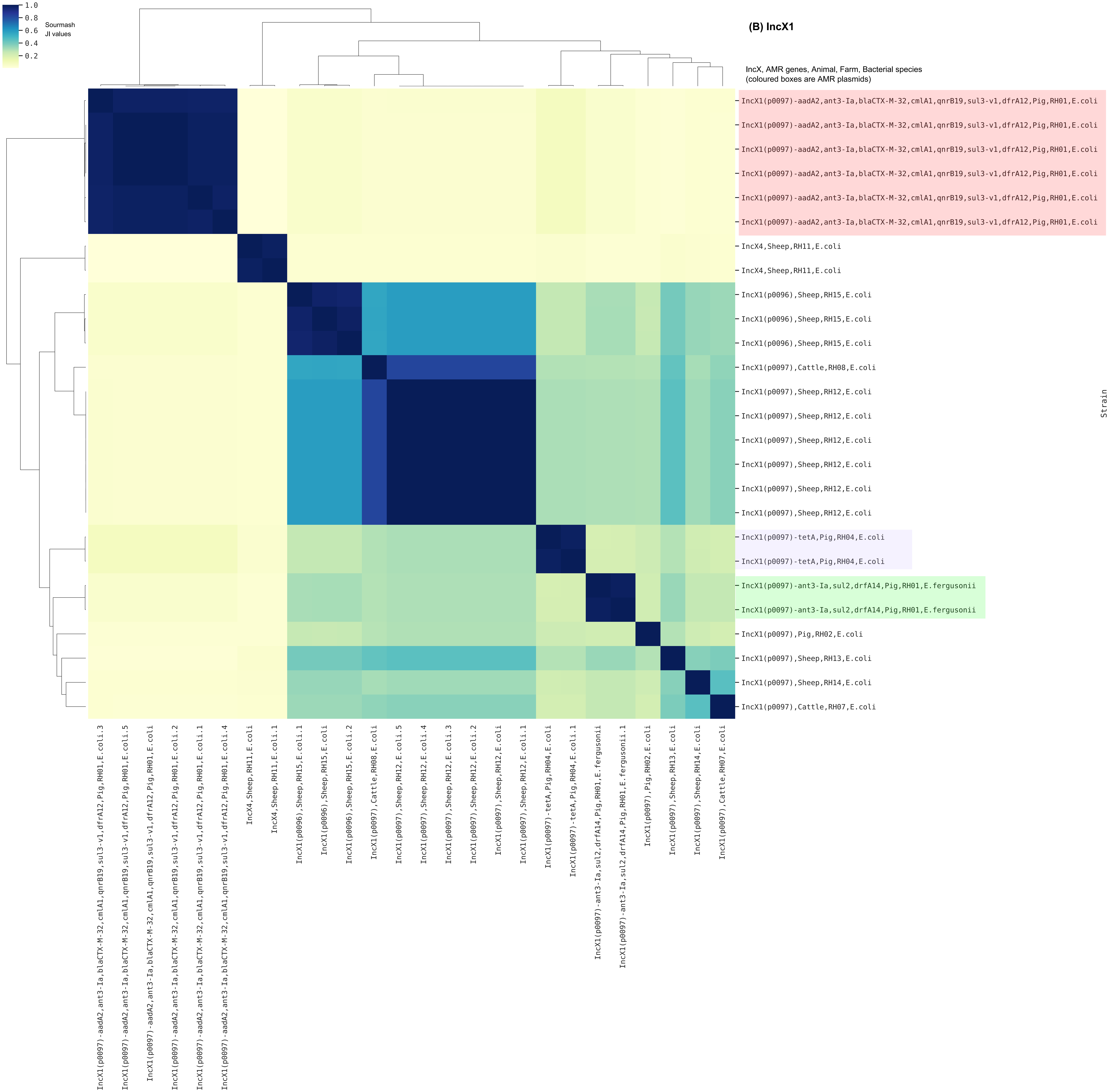
1.0
0.8
0.6
0.4
0.2
Sourmash
JI values
(B) IncX1
IncX, AMR genes, Animal, Farm, Bacterial species
(coloured boxes are AMR plasmids)
IncX1(p0097)-aadA2,ant3-Ia,blaCTX-M-32,cmlA1,qnrB19,sul3-v1,dfrA12,Pig,RH01,E.coli
IncX4,Sheep,RH11,E.coli
IncX1(p0096),Sheep,RH15,E.coli
IncX1(p0097),Cattle,RH08,E.coli
IncX1(p0097),Sheep,RH12,E.coli
IncX1(p0097)-tetA,Pig,RH04,E.coli
IncX1(p0097)-ant3-Ia,sul2,drfA14,Pig,RH01,E.fergusonii
IncX1(p0097),Pig,RH02,E.coli
IncX1(p0097),Sheep,RH13,E.coli
IncX1(p0097),Sheep,RH14,E.coli
IncX1(p0097),Cattle,RH07,E.coli
Strain

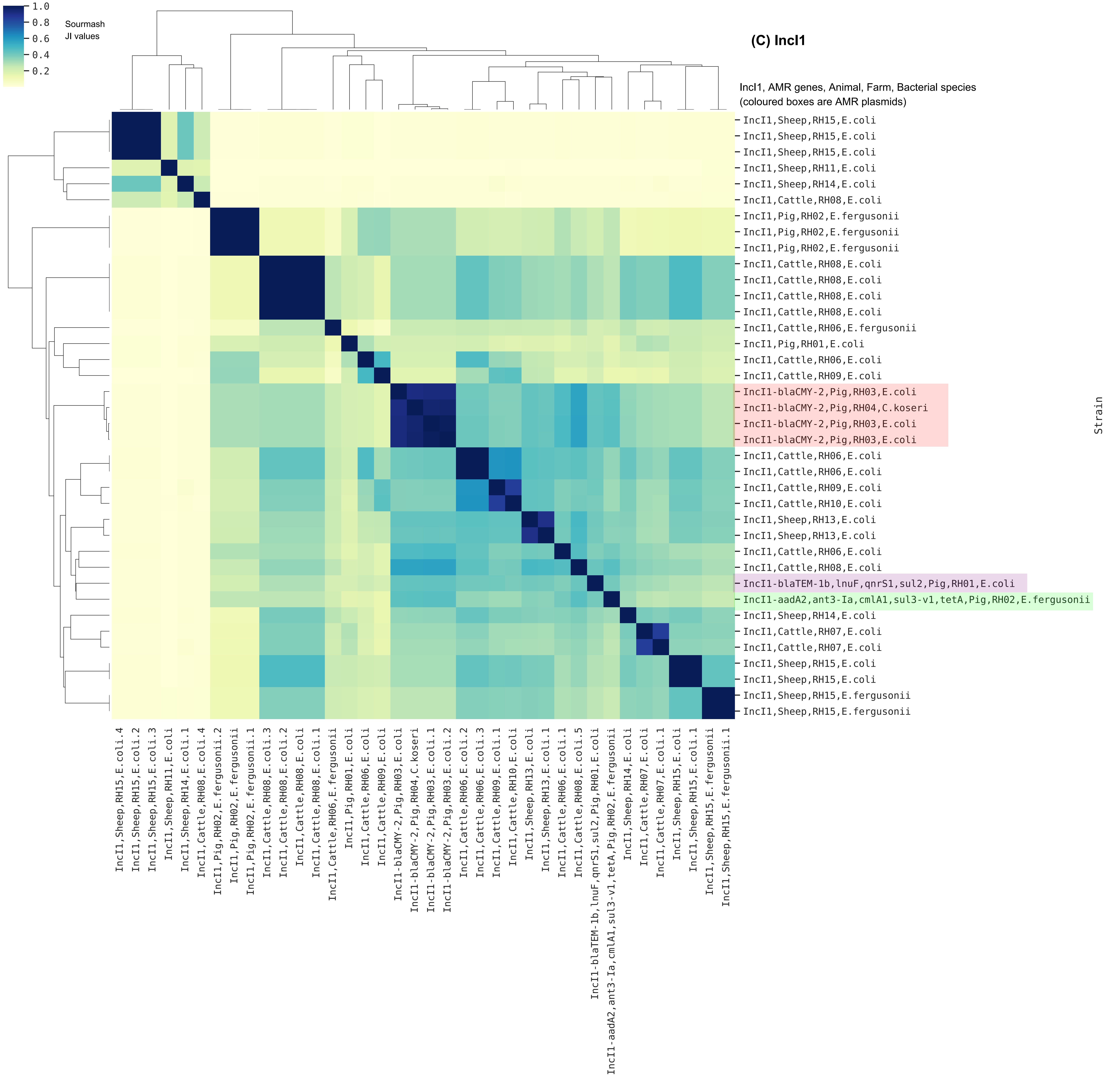

Sourmash
JI values
1.0
0.8
0.6
0.4
0.2
(C) IncI1
IncI1, AMR genes, Animal, Farm, Bacterial species
(coloured boxes are AMR plasmids)
IncI1,Sheep,RH15,E.coli
IncI1,Sheep,RH15,E.coli
IncI1,Sheep,RH15,E.coli
IncI1,Sheep,RH11,E.coli
IncI1,Sheep,RH14,E.coli
IncI1,Cattle,RH08,E.coli
IncI1,Pig,RH02,E.fergusonii
IncI1,Pig,RH02,E.fergusonii
IncI1,Pig,RH02,E.fergusonii
IncI1,Cattle,RH08,E.coli
IncI1,Cattle,RH08,E.coli
IncI1,Cattle,RH08,E.coli
IncI1,Cattle,RH08,E.coli
IncI1,Cattle,RH06,E.fergusonii
IncI1,Pig,RH01,E.coli
IncI1,Cattle,RH06,E.coli
IncI1,Cattle,RH09,E.coli
IncI1-blaCMY-2,Pig,RH03,E.coli
IncI1-blaCMY-2,Pig,RH04,C.koseri
IncI1-blaCMY-2,Pig,RH03,E.coli
IncI1-blaCMY-2,Pig,RH03,E.coli
IncI1,Cattle,RH06,E.coli
IncI1,Cattle,RH06,E.coli
IncI1,Cattle,RH09,E.coli
IncI1,Cattle,RH10,E.coli
IncI1,Sheep,RH13,E.coli
IncI1,Sheep,RH13,E.coli
IncI1,Cattle,RH06,E.coli
IncI1,Cattle,RH08,E.coli
IncI1-blaTEM-1b,lnuF,qnrS1,sul2,Pig,RH01,E.coli
IncI1-aadA2,ant3-Ia,cmlA1,sul3-v1,tetA,Pig,RH02,E.fergusonii
IncI1,Sheep,RH14,E.coli
IncI1,Cattle,RH07,E.coli
IncI1,Cattle,RH07,E.coli
IncI1,Sheep,RH15,E.coli
IncI1,Sheep,RH15,E.coli
IncI1,Sheep,RH15,E.fergusonii
IncI1,Sheep,RH15,E.fergusonii
Strain
IncI1,Sheep,RH15,E.coli.4
IncI1,Sheep,RH15,E.coli.2
IncI1,Sheep,RH15,E.coli.3
IncI1,Sheep,RH11,E.coli
IncI1,Sheep,RH14,E.coli.1
IncI1,Cattle,RH08,E.coli.4
IncI1,Pig,RH02,E.fergusonii.2
IncI1,Pig,RH02,E.fergusonii
IncI1,Pig,RH02,E.fergusonii.1
IncI1,Cattle,RH08,E.coli.3
IncI1,Cattle,RH08,E.coli.2
IncI1,Cattle,RH08,E.coli
IncI1,Cattle,RH08,E.coli.1
IncI1,Cattle,RH06,E.fergusonii
IncI1,Pig,RH01,E.coli
IncI1,Cattle,RH06,E.coli
IncI1,Cattle,RH09,E.coli
IncI1-blaCMY-2,Pig,RH03,E.coli
IncI1-blaCMY-2,Pig,RH04,C.koseri
IncI1-blaCMY-2,Pig,RH03,E.coli.1
IncI1-blaCMY-2,Pig,RH03,E.coli.2
IncI1,Cattle,RH06,E.coli.2
IncI1,Cattle,RH06,E.coli.3
IncI1,Cattle,RH09,E.coli.1
IncI1,Cattle,RH10,E.coli
IncI1,Sheep,RH13,E.coli
IncI1,Sheep,RH13,E.coli.1
IncI1,Cattle,RH06,E.coli.1
IncI1,Cattle,RH08,E.coli.5
IncI1-blaTEM-1b,lnuF,qnrS1,sul2,Pig,RH01,E.coli
IncI1-aadA2,ant3-Ia,cmlA1,sul3-v1,tetA,Pig,RH02,E.fergusonii
IncI1,Sheep,RH14,E.coli
IncI1,Cattle,RH07,E.coli
IncI1,Cattle,RH07,E.coli.1
IncI1,Sheep,RH15,E.coli
IncI1,Sheep,RH15,E.coli.1
IncI1,Sheep,RH15,E.fergusonii
IncI1,Sheep,RH15,E.fergusonii.1
