# Supplementary material for: A genomic epidemiological study shows that prevalence of antimicrobial resistance in Enterobacterales is associated with the livestock host, as well as antimicrobial usage
Source: Microb Genom. 2021 Oct 5;7(10):000630. doi: 10.1099/mgen.0.000630 (PMC8627209; doi:10.1099/mgen.0.000630)
Supplement: Supplementary material 3 [file mgen-7-0630-s003.pdf]

S2 Table. Summary of isolates recovered from 14 farms

**Recovered on**	**Animal Species**	**Number of farms**	**Total number of isolates**			***C. koseri***		***E. coli***		***E. fergusonii***		***K. pneumoniae***	
			Total Number	Number with AMR genes	Number of MDR*	Total Number	Number of MDR*	Total Number	Number of MDR*	Total Number	Number of MDR*	Total Number	Number of MDR*
No antibiotic	Cattle	5	55	10	2	5 (5)		49	1	1			
	Pig	4	35	25	16	3 (2)	1	24	7	6	6	2 (2)	
	Sheep	5	57	7				55		2			
1mg/L Cefotaxime	Cattle	1	3	3	3			3	3				
	Pig	3	18	17	14			18	14				
	Sheep												
Total Number			168	62	35	8	2	149	25	9	6	2	2

*The number of MDR isolates, harbouring three or more AMR classes.

The numbers in brackets indicate the number of isolates harbouring intrinsic AMR genes.

S3 Table. Summary of the antimicrobial classes (mg/kg and total mg) used by each farm.

Antimicrobial class	Antimicrobial Usage (mg/kg)										Total Antimicrobial Usage (mg in 100s)									
	Pig			Cattle					Sheep		Pig			Cattle					Sheep	
	RH01	RH02	RH04	RH06	RH07	RH08	RH09	RH10	RH12	RH14	RH01	RH02	RH04	RH06	RH07	RH08	RH09	RH10	RH12	RH14
Aminoglycoside	18.6	28.6		60.3	31.6		113.7	19.3			16000	6750		268.8	324.1		1450	82.1		
ESCs[1]					3.2	10.6	18.7	8.6							90	45	154.5	36.75		
Non-ESC beta-lactams[1]	32.5	44.1	17.2	223.7	32.4		166.3	21.5			35494	4750	411.6	4536	525.35		3139	91.25		
Macrolides	93.1	139.5	8.0		11.8		122.4	39.0			151630	23625	161.2		100		840	155.3		
Quinolones	7.5	7.5				0.6	14.1	8.0			150	37.5				5	60	34		
Sulphonamide	699.9						150.6				1123662.5						1120			
Tetracycline	48.2	143.1				70.6	53.6	10.6	1.9	13.3	56790	32200				300	75	45	20	60
Trimethoprim	140.0						30.1				224732.5						224			
**Grand Total**	**1039.8**	**362.8**	**25.2**	**284.0**	**78.9**	**81.8**	**669.5**	**107.1**	**1.9**	**13.3**	**1608459**	**67362.5**	**572.8**	**4804.8**	**1041.5**	**350**	**7062.5**	**453.4**	**20**	**60**

[1]ESC = Extended spectrum cephalosporins

S4 Table. Results of individual multivariable mixed effects logistic regression analysis for each antimicrobial resistance gene presence for an antimicrobial classes and associations with farm usage of all and corresponding antimicrobial classes (weight of active ingredient used). Farm ID was included as a random effect.

**Antimicrobial class of gene presence**	**Number of isolates carrying specific class AMR gene (%)[1]**	**Total farm usage of corresponding antimicrobial class (per 10g)**			**Total farm usage of all antimicrobial classes (per 100g)**		
		Odds Ratio	95% Confidence Interval	P Value	Odds Ratio	95% Confidence Interval	P Value
Aminoglycosides	19 (13.1)	1.159	0.981-1.370	0.083	1.241	0.825-1.868	0.300
Non-ESC[2] beta-lactams	8 (5.5)	1.013	1.001-1.026	0.031	1.003	1.001-1.005	0.005
Phenicols[3]	7 (4.8)	-	-	-	1.005	0.999-1.012	0.093
Quinolones	8 (5.5)	18.352	1.202-280.077	0.036	1.002	0.999-1.005	0.303
Sulphonamides	17 (11.7)	1.001	1.000-1.001	0.068	1.004	1.000-1.008	0.051
Tetracycline	29 (20.0)	1.050	0.898-1.227	0.539	1.245	0.799-1.939	0.332
Trimethoprim	8 (5.5)	1.003	1.001-1.005	0.011	1.004	1.001-1.007	0.008
Multi-drug resistance[4]	15 (10.3)	-	-	-	1.004	1.000-1.009	0.057

[1]N=145, [2]ESC = Extended spectrum cephalosporins, [3]No on-farm usage of phenicols in the three month period before sampling, [4]Isolates were considered multi-drug resistant if they harboured resistance genes to three or more antimicrobial classes
